# Laboratory and Field Investigation on the Damp-Heat Aging Resistance of Steel Slag Asphalt Pavement

**DOI:** 10.3390/ma19214633

**Published:** 2026-10-31

**Authors:** Liangliang Tu, Yu Song, Xinkui Yang, Shaopeng Wu, Jianlin Feng, Haiqin Xu

**Affiliations:** 1 State Key Laboratory of Silicate Materials for Architectures, Wuhan University of Technology, Wuhan 430070, China; tlliang@whut.edu.cn (L.T.);; 2 Shenzhen Sez Construction Group Co., Ltd., Shenzhen 518034, China

**Keywords:** asphalt pavement, damp-heat aging, steel slag asphalt mixture, rheological property, road performance

## Abstract

Damp-heat environments significantly accelerate the aging and performance deterioration of asphalt pavements. This study systematically evaluated the performance evolution of base and SBS-modified asphalt binders, as well as their corresponding steel slag and basalt asphalt mixtures, under diverse damp-heat aging conditions (60–80 °C, 90% RH, 0–8 days). Physical and rheological characterizations indicate that base asphalt exhibits a monotonic hardening law during damp-heat aging, whereas SBS-modified asphalt demonstrates a unique two-stage aging process characterized by initial softening followed by hardening. Chemical functional group analysis further confirms that damp-heat aging results from the synergistic effects of early-stage moisture ingress and long-term thermal-oxidative hardening—a process effectively delayed by the SBS polymer network. Furthermore, microscopic morphology and elemental analysis reveal that steel slag aggregates establish a robust dual mechanism to resist moisture erosion through the mechanical interlocking of surface micropores and the chemical anchoring of alkaline constituents. A comprehensive evaluation using the improved radar chart method (f-value) further substantiates that steel slag not only enhances the initial performance of the mixtures but also significantly reduces their time–temperature sensitivity to damp-heat environments. Finally, full-scale field applications in damp-heat regions demonstrate that steel slag asphalt pavements maintain a residual Marshall stability of 87.6% and a texture depth retention rate of 91.5% after service. These results indicate superior early-to-mid-term moisture damage resistance and safety compared to basalt-based pavements, providing critical theoretical and practical support for material design and performance improvement in extreme damp-heat climatic regions.

## 1. Introduction

Asphalt concrete is the predominant paving material for highway infrastructure worldwide; however, its long-term serviceability is persistently challenged by complex environmental weathering [1,2,3]. At the core of this material is the asphalt binder, a fundamental temperature-sensitive polymer that relies on high fluidity at elevated temperatures to facilitate construction, while requiring adequate mechanical strength at ambient conditions to resist deformation. To further enhance pavement durability, styrene-butadiene-styrene (SBS) modified asphalt is extensively utilized in high-grade pavements owing to its superior adhesion and crack-resistance properties [4,5,6]. However, during pavement service, combined environmental stressors—such as ultraviolet radiation, oxygen, heat, and moisture—accelerate the aging of asphalt binders, leading to a progressive deterioration of their mechanical and rheological properties [7,8,9,10].

Among various climatic stressors, damp-heat environments—characterized by elevated ambient temperatures coupled with intense humidity or abundant rainfall—impose exceptionally severe coupled damage on asphalt pavements. The synergistic effect of thermal-oxidative aging and pervasive moisture intrusion drastically weakens the asphalt-aggregate interfacial adhesion, triggering premature distresses such as potholes, raveling, and structural cracking. These distresses not only pose severe safety risks to road users but also significantly increase maintenance costs [11,12]. Although SBS modification mitigates these issues to some extent, prolonged exposure to extreme damp-heat conditions can still disrupt its reinforcing polymer network and accelerate interfacial stripping. While extensive studies have investigated thermal-oxidative aging [13,14], PAV-simulated aging, and UV-induced aging of asphalt materials, these standardized testing protocols typically isolate environmental variables and often fail to accurately simulate the complex moisture permeation and chemical erosion that occur under prolonged, extreme damp-heat conditions. Specifically, the fundamental coupled degradation mechanism—where intense moisture intrusion continuously disrupts the asphalt-aggregate interface while elevated temperatures concurrently accelerate binder oxidation—remains poorly understood. Therefore, there is a critical need to bridge this research gap by systematically investigating the performance evolution and interfacial failure mechanisms of asphalt mixtures under severe coupled damp-heat conditions to inform the design and maintenance of asphalt pavements in hot-humid climates.

In regions with high ambient temperature and humidity, the performance degradation of asphalt binders under the combined effects of heat and moisture is inevitable. According to the Technical Specifications for Construction of Highway Asphalt Pavements in China [15], areas with an annual rainfall exceeding 1000 mm are classified as humid regions, where the requirements for the moisture stability of asphalt mixtures are the most stringent. Specifically, the residual stability of the immersion Marshall test and the residual tensile strength ratio of polymer-modified asphalt mixtures must not be lower than 85% and 80%, respectively. These requirements arise mainly because moisture can penetrate the asphalt pavement structure, reducing the adhesion between asphalt and aggregates and causing debonding [16]. Moreover, the asphalt film coating the aggregate surface may be eroded and stripped by water, leading to aggregate exposure and a consequent reduction in the structural stability of the asphalt concrete. Lu et al. [17] evaluated the Marshall stability of asphalt mixtures subjected to different numbers of wet–dry cycles and found that such cycling significantly deteriorates the adhesion between asphalt and aggregates. After 24 wet–dry cycles at 60 °C, the Marshall stability decreased by more than 50%. Asphalt mixtures with higher air void content are particularly vulnerable to moisture intrusion, resulting in more severe moisture-induced damage in asphalt pavements [18,19].

Moisture not only erodes the asphalt–aggregate interface in mixtures but may also interact with polar compounds in asphalt or its oxidation products, thereby introducing complex effects on thermal–oxidative aging. Liu et al. [20] reported that the free-radical oxidation mechanism of asphalt involves three stages: initiation through hydrogen abstraction by reactive species, propagation through oxygen addition that promotes chain growth, and termination via the decomposition of hydroperoxides through hydrogen migration, ultimately forming carbonyl and other oxygenated products. Elevated temperatures intensify molecular motion and significantly accelerate the rate of thermal–oxidative aging. Ma et al. [21] further demonstrated that increasing temperature markedly enhances asphalt oxidation and thermal degradation by increasing molecular kinetic energy, resulting in substantial growth of the carbonyl and sulfoxide indices, whereas higher humidity causes only slight increases in these indices. The underlying mechanism is that high-temperature steam induces an oxygen-absorbing oxidation process in which hydrophilic groups migrate toward interfaces through hydrogen-bond interactions, promoting the formation of oxygen-containing functional groups. Elevated temperatures render asphalt molecules more reactive and unstable, facilitating the generation of free radicals and accelerating aging. Meanwhile, water molecules can hydrate polar species in asphalt, further promoting oxidative reactions. Thus, the combined effects of high temperature and moisture on the aging behavior of asphalt binders are complex and cannot be neglected.

Furthermore, the durability of asphalt mixtures in damp-heat environments is heavily dictated by the interfacial adhesion between the asphalt binder and aggregates [22,23]. Unlike traditional acidic aggregates such as basalt, which are highly susceptible to moisture-induced stripping, steel slag—a widely available industrial solid waste—has demonstrated significant potential as an alternative aggregate [24]. However, although the anti-stripping benefits of steel slag are generally recognized, a critical unresolved scientific question remains: the long-term performance evolution of steel slag and basalt asphalt mixtures under different damp-heat environments, and the physicochemical mechanisms by which the alkaline surface chemistry and highly porous micro-texture of steel slag—relative to conventional acidic and dense basalt aggregates—mitigate moisture-driven interfacial degradation during long-term aging.

The physical properties of asphalt—including penetration, ductility, softening point, and Brookfield viscosity—reflect its hardness, crack resistance, and high-temperature stability, and are commonly used to evaluate the degree of aging [25]. Aging also significantly alters the rheological behavior of asphalt binders. Zhang et al. [26] examined the effects of thermal-oxidative aging on the rheological properties of SBS-modified asphalt and found that indicators such as complex modulus, phase angle, and non-recoverable compliance exhibited strong correlations with the extent of aging. In addition to macroscopic physical and rheological properties, Fourier-transform infrared spectroscopy (FTIR) is widely employed to investigate asphalt aging mechanisms, as it can identify chemical functional groups formed or diminished during aging. By quantifying the absorbance of specific functional groups, FTIR enables a more precise assessment of aging severity in asphalt binders [27].

**Figure 1:**
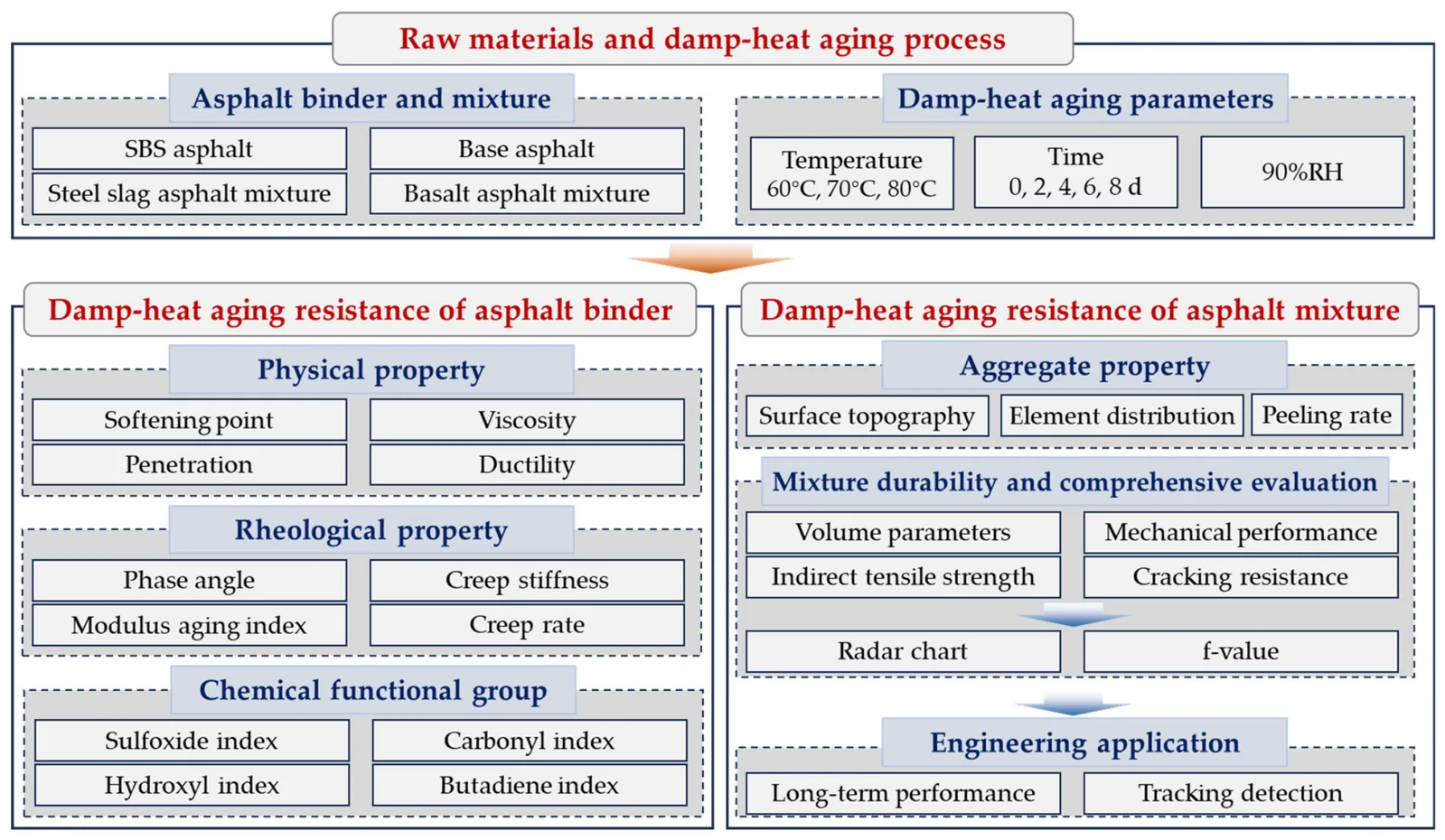
The technical roadmap.

This study aims to investigate the performance of steel slag and basalt asphalt mixtures under different damp-heat aging conditions. The novelty of this work lies in revealing that the effect of damp-heat aging on asphalt mixture performance follows the time–temperature superposition principle, which facilitates the prediction of long-term durability. Additionally, the mechanisms by which steel slag aggregate enhances the damp-heat aging resistance of asphalt mixtures relative to basalt aggregate were identified. Specifically, the physical properties, rheological behavior, and chemical functional groups of SBS-modified and base asphalt binders were first evaluated under diverse damp-heat aging conditions (60, 70, and 80 °C, 90% RH for 0–8 days). Subsequently, the microscopic morphology, elemental distribution, and interfacial adhesion of steel slag and basalt aggregates were comparatively analyzed. The study further evaluated the macroscopic road performances of steel slag and basalt asphalt mixtures, including Marshall stability, indirect tensile strength, and low-temperature cracking resistance. A unique comprehensive performance index (f-value) based on an improved radar chart was introduced to quantitatively assess the damp-heat aging resistance of the mixtures. Finally, a full-scale field test section in a typical damp-heat region was continuously monitored for two years to validate the long-term durability of the steel slag asphalt pavement. The technical roadmap of this study is illustrated in Figure 1.

## 2. Materials and Methods

### 2.1. Raw Materials

The materials utilized in this study included penetration grade 60/80 (Pen. 60/80) asphalt, styrene-butadiene-styrene (SBS) modified asphalt, steel slag aggregates, basalt aggregates, and mineral filler, all sourced from Guangdong Province, China. The technical properties of these materials are detailed in Table 1, Table 2 and Table 3.

### 2.2. Design of Asphalt Mixture

The widely adopted AC-13 gradation was utilized to design the steel slag and basalt asphalt concrete mixtures. For the basalt mixture, basalt aggregate and limestone mineral powder were adopted as the raw materials. For the steel slag mixture, steel slag accounted for 100% of the aggregate blend by mass. Both mixtures were designed with the Technical Specification for Construction of Highway Asphalt Pavements (JTG F40-2004) [28] and proportioned by mass according to the median values of the AC-13 gradation, and the resulting aggregate gradations were kept closely comparable. The SBS asphalt content was fixed at 4.8% for both mixtures to ensure that subsequent comparisons of mixture performance before and after damp-heat aging were based on consistent binder content and gradation. This design strategy was adopted to create a clear contrast between the damp-heat aging resistance of steel slag and the basalt aggregate.

**Figure 2:**
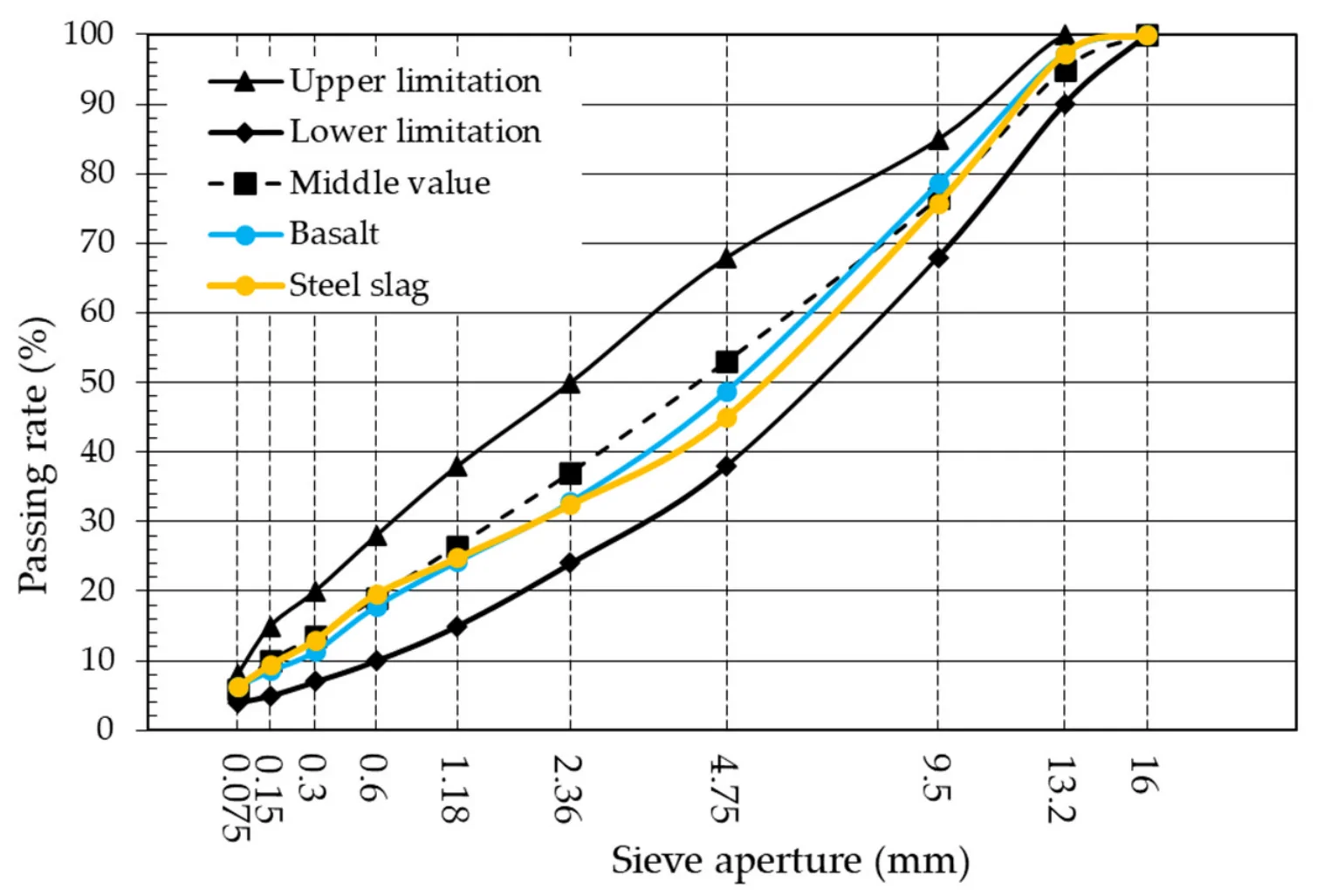
The aggregate gradation curve of steel slag and basalt asphalt mixture.

The detailed aggregate gradation information is shown in Table 4, aggregate compositions of the two mixtures are listed in Table 5, the proportions of each aggregate fraction are presented in Table 6, and the corresponding gradation curves are illustrated in Figure 2. Both two asphalt mixtures are prepared according to the Standard Test Methods of Asphalt and Asphalt Mixture for Highway Engineering (JTG 3410-2025) [29]. The aggregates and the SBS asphalt binder were preheated at 180 °C and 165 °C for 5 h, respectively. Subsequently, they were mixed at 175 °C and compacted at 165 °C using a Marshall compactor with 75 blows per face. The volumetric parameters of the two mixtures are summarized in Table 7.

### 2.3. Damp-Heat Aging Method

**Figure 3:**
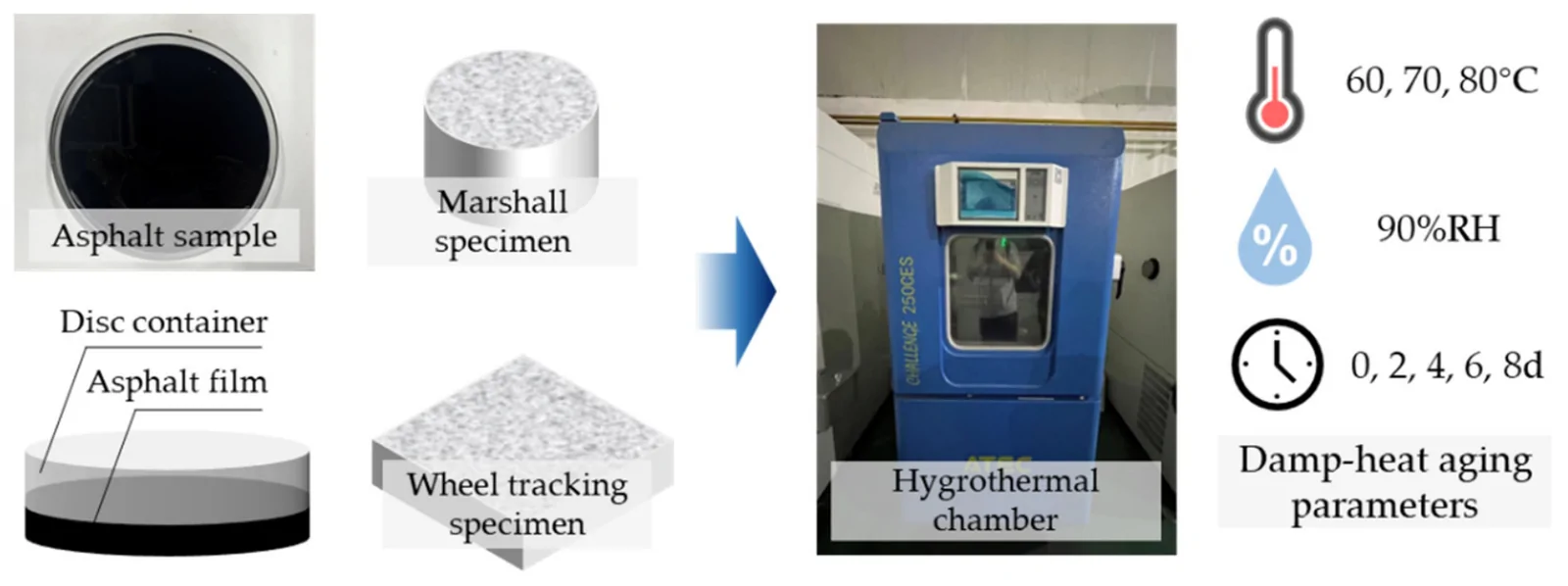
Damp-heat test of asphalt film and mixture sample.

The asphalt samples, concrete samples, and the high–low temperature damp-heat chamber (ATEC, SU series) used to simulate their damp-heat aging are shown in Figure 3. In high-temperature and high-humidity regions during summer, asphalt pavements may experience relative humidity levels of up to 90% RH and surface temperatures reaching 70 °C. Accordingly, a relative humidity of 90% RH and temperatures of 60, 70, and 80 °C were selected to simulate representative damp-heat aging conditions. In contrast to the traditional soaking procedures applied to asphalt or asphalt mixture samples in most previous studies, this research utilized a custom damp-heat aging environmental chamber. By precisely controlling both temperature and humidity, this apparatus simulates damp-heat conditions that more accurately reflect actual field environments. The specific damp-heat aging parameters and sample designations for the asphalt binders and asphalt concrete are detailed in Table 8 and Table 9, respectively. In Table 8, the prefixes “B” and “S” refer to base asphalt and SBS-modified asphalt samples, respectively. In Table 9, “BM” and “SM” refer to base asphalt mixture and SBS-modified asphalt mixture, respectively.

### 2.4. Performance Testing of Asphalt Binders, Aggregates, and Mixtures

**Figure 4:**
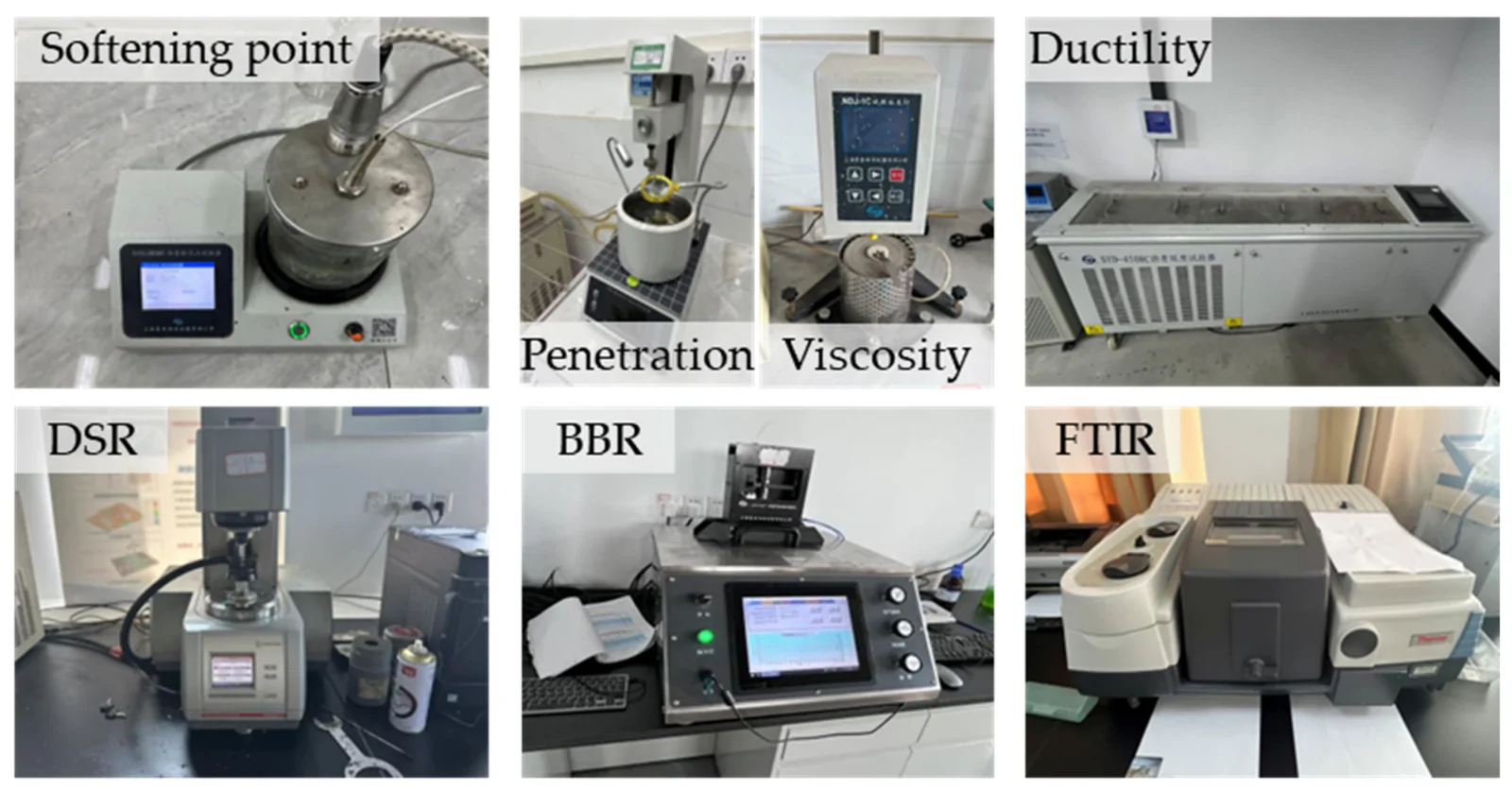
Experiments of physical, rheological property and chemical functional group.

The effects of damp-heat aging on the performance of SBS-modified asphalt and base asphalt were evaluated through tests of physical properties, rheological characteristics, and chemical functional group structures. The mechanical properties, cohesive strength, and low-temperature cracking resistance of both the steel slag and basalt asphalt mixtures were evaluated using Marshall stability tests, indirect tensile tests, and beam bending tests. The equipment used for these tests are shown in Figure 4.

#### 2.4.1. Damp-Heat Aging Resistance of Asphalt Binders

(1) Physical performance

The penetration, ductility, softening point and Brookfield viscosity of asphalt samples under different damp-heat aging conditions were tested according to the standard test method of asphalt and asphalt mixture for highway engineering, and its low-temperature flexibility and high-temperature stability were studied. The retention rate of penetration (RRP) and ductility (RRD), increment of softening point (ISP), and viscosity aging index (VAI), are used to evaluate the effect of damp-heat aging on asphalt binders. The RRP, RRD, ISP, and VAI were calculated by Equations (1)–(4), respectively.

(1) $$RRP=\frac{P_{2}}{P_{1}}\times 100\%$$

(2) $$RRD=\frac{D_{2}}{D_{1}}\times 100\%$$

(3) $$ISP={SP}_{2}-{SP}_{1}$$

(4) $$VAI=\frac{V_{2}-V_{1}}{V_{1}}\times 100\%$$

where $P_{1}$, $D_{1}$, ${SP}_{1}$, and $V_{1}$ represent the penetration, ductility, softening point, and viscosity of asphalt before damp-heat aging; $P_{2}$, $D_{2}$, ${SP}_{2}$, and $V_{2}$ represent those of asphalt after damp-heat aging.

(2) Rheological performance

The rheological properties of the asphalt binders under various temperature conditions were systematically characterized using a dynamic shear rheometer (DSR) and a bending beam rheometer (BBR). The DSR tests were conducted in temperature-controlled mode, with a temperature sweep performed from 30 °C to 80 °C at a heating rate of 1.5 °C/min and a fixed frequency of 10 rad/s. The resulting variations in complex modulus (G^*^) and phase angle (δ) with temperature were used to evaluate the high-temperature performance of the binders. The BBR tests were carried out at low temperatures of −12 °C and −18 °C. By measuring the creep stiffness (S) and creep rate (m-value) of asphalt beam specimens under constant loading, the low-temperature cracking resistance and stress relaxation capability were assessed. In addition, the degree of damp-heat aging of each binder was evaluated using the modulus aging index (MAI), calculated from the initial (30 °C) complex modulus in the DSR test, as defined in Equation (5) [30].

(5) $$\begin{array}{c} \mathrm{MAI}=\frac{G_{2}-G_{1}}{G_{1}}\times 100\% \end{array}$$

where $G_{1}$ denotes the initial complex modulus of unaged asphalt binder, and $G_{2}$ represents the initial complex modulus of aged asphalt binder under different damp-heat aging conditions.

(3) Chemical functional group

Fourier-transform infrared spectroscopy (Nicolet iS50 FTIR, Shanghai, China) was employed to identify the characteristic functional groups in asphalt and their relative contents [31,32]. The chemical structures of asphalt samples before and after damp-heat aging were characterized using FTIR. Samples were dissolved in carbon disulfide and then applied onto potassium bromide (KBr) crystal surfaces. After the solvent was evaporated, FTIR measurements were conducted over a wavenumber range of 4000–400 cm^−1^. The degree of damp-heat aging was quantitatively assessed using the carbonyl index, sulfoxide index, hydroxyl, and butadiene index, calculated according to Equations (6)–(9) respectively [33].

(6) $$\begin{array}{c} I_{C=O}=\frac{A_{1700}}{A_{1374}}\times 100\% \end{array}$$

(7) $$\begin{array}{c} I_{S=O}=\frac{A_{1030}}{A_{1374}}\times 100\% \end{array}$$

(8) $$\begin{array}{c} I_{O-H}=\frac{A_{3300}}{A_{1374}}\times 100\% \end{array}$$

(9) $$\begin{array}{c} I_{B}=\frac{A_{966}}{A_{1374}}\times 100\% \end{array}$$

Here, $A_{1700}$ represents the peak area of the carbonyl characteristic absorption at 1700 cm^−1^, and $A_{1374}$ corresponds to the peak area of the saturated hydrocarbon bond vibration at 1374 cm^−1^. $A_{1030}$ denotes the peak area of the sulfoxide characteristic absorption at 1030 cm^−1^, while $A_{3300}$ represents the peak area of the hydroxyl characteristic absorption at 3300 cm^−1^. $A_{966}$ represents the butadiene absorption peak area at 966 cm^−1^.

#### 2.4.2. Performance Characterization of Steel Slag Aggregate

(1) Surface micro-morphology and element distribution

The surface morphology and elemental distribution of the aggregates were characterized using a scanning electron microscope equipped with an energy dispersive X-ray spectrometer (SEM-EDS). Prior to examination, the aggregate samples were thoroughly cleaned, dried, and sputter-coated with gold to enhance their electrical conductivity. Morphological observations were conducted utilizing a JSM-IT800 (JEOL Ltd., Kyoto, Japan) field emission scanning electron microscope (FE-SEM) operating at an accelerating voltage of 15 kV. Additionally, elemental mapping was performed to analyze the regional composition, thereby elucidating the surface microstructural and chemical characteristics of the aggregates.

(2) Adhesion to asphalt

The adhesive performance between asphalt and aggregates was evaluated using an improved boiling water test. Dried coarse aggregates (10–16 mm) were fixed with thin iron wires, heated to 175 °C, and uniformly coated with asphalt heated to 165 °C. After cooling, the adhesive performance was assessed based on the mass loss after boiling for 10 min, as calculated by Equation (10):

(10) $$\begin{array}{c} \mathrm{Peeling}\;\mathrm{rate}=\frac{m_{1}-m_{2}}{m_{1}}\times 100\% \end{array}$$

where $m_{1}$ and $m_{2}$ represent the mass of the asphalt-coated aggregate before and after boiling, respectively.

#### 2.4.3. Comprehensive Evaluation on Damp-Heat Aging Resistance of Asphalt Mixtures

In this study, a uniquely modified radar chart evaluation method was employed to quantitatively assess the comprehensive performance of asphalt mixtures under damp-heat aging conditions. Initially, a multidimensional evaluation index matrix, denoted as $A={\left(a_{ij}\right)}_{n\times k}$, was constructed. This matrix incorporates key performance parameters derived from standardized testing protocols, including residual Marshall stability (RMS), tensile strength ratio (TSR), and low-temperature maximum flexural strain. Subsequent to data collection, the raw data were subjected to standardization and nonlinear transformation. This procedure effectively eliminates dimensional discrepancies and amplifies the distinguishability among the evaluated indicators. Following this, the geometric feature vectors of the radar chart, specifically the characteristic area ($A_{i}$) and characteristic perimeter ($L_{i}$), were extracted. These features were utilized to construct a normalized evaluation vector, $v_{i}=[v_{i1},v_{i2}]$, where $v_{i1}$ and $v_{i2}$ represent the relative area and relative perimeter, respectively. Ultimately, a unique comprehensive performance index, f, was derived using the evaluation function $f(v_{i1},v_{i2})=\sqrt{v_{i1}\times v_{i2}}$. The index f ranges from 0 to 1; a higher value signifies a more balanced retention of material properties and superior comprehensive durability under damp-heat aging conditions. Consequently, this index facilitates an objective ranking and quantitative comparison of the aging resistance across various aggregate and asphalt combinations.

## 3. Effect of Damp-Heat Aging on Asphalt Binder

### 3.1. Macroscopic Morphology of SBS-Modified Asphalt Before and After Damp-Heat Aging

**Figure 5:**
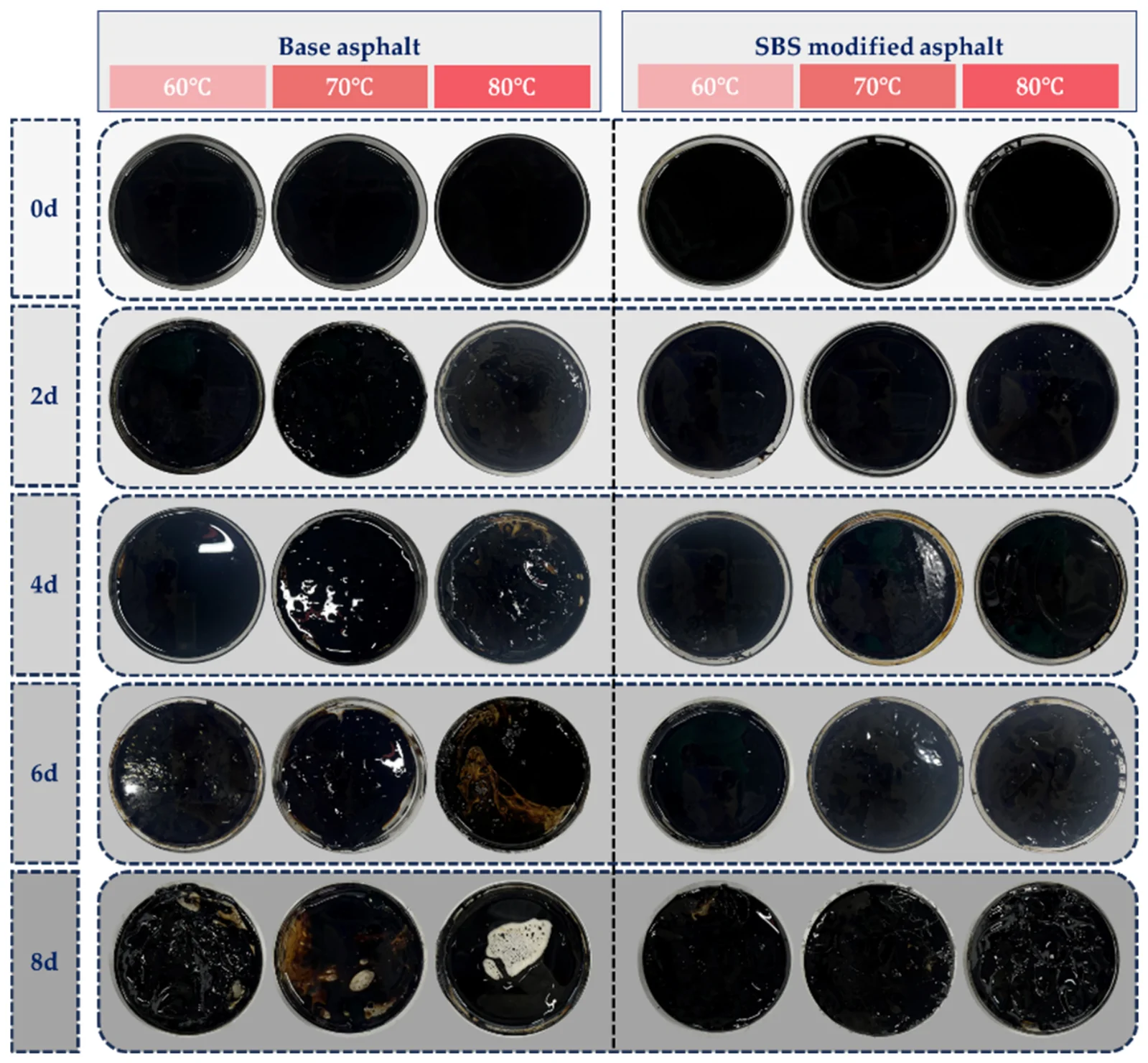
Asphalt morphology under different damp-heat aging conditions.

Figure 5 illustrates the morphological evolution of base asphalt and SBS-modified asphalt during damp-heat aging over time. As the aging temperature and duration increased, the macroscopic surfaces of both asphalt types gradually became rougher and lost gloss, with color changing from deep black to brownish hues. Under the high-humidity conditions provided by the damp-heat aging chamber, water condensed on the asphalt surfaces, forming visible liquid accumulation, closely replicating real-world conditions. In the early stage of damp-heat aging (0–2 days), both asphalt types maintained an intact black surface; however, the base asphalt exhibited slight loss of gloss by day 2, whereas the SBS-modified asphalt retained good surface shine. After 4 days of aging, the base asphalt surface showed oil bleeding in the condensed water, particularly for samples aged at 80 °C, while the SBS-modified asphalt only exhibited increased surface roughness and slight local lightening of color, maintaining overall structural integrity. By the late stage of aging (8 days), prolonged exposure to damp-heat conditions caused pore formation on the base asphalt surface due to moisture accumulation and extrusion, with higher temperatures exacerbating this effect. In contrast, SBS-modified asphalt began to exhibit oil bleeding only after day 4, and even at 80 °C, no significant macroscopic pores were observed. This enhanced performance is attributed to the reinforcing SBS polymer network, which improves the asphalt’s resistance to high-temperature deformation and water-induced damage.

### 3.2. Physical Properties of SBS-Modified Asphalt

#### 3.2.1. Effects of Damp-Heat Aging on the High-Temperature Performance of SBS-Modified Asphalt

**Figure 6:**
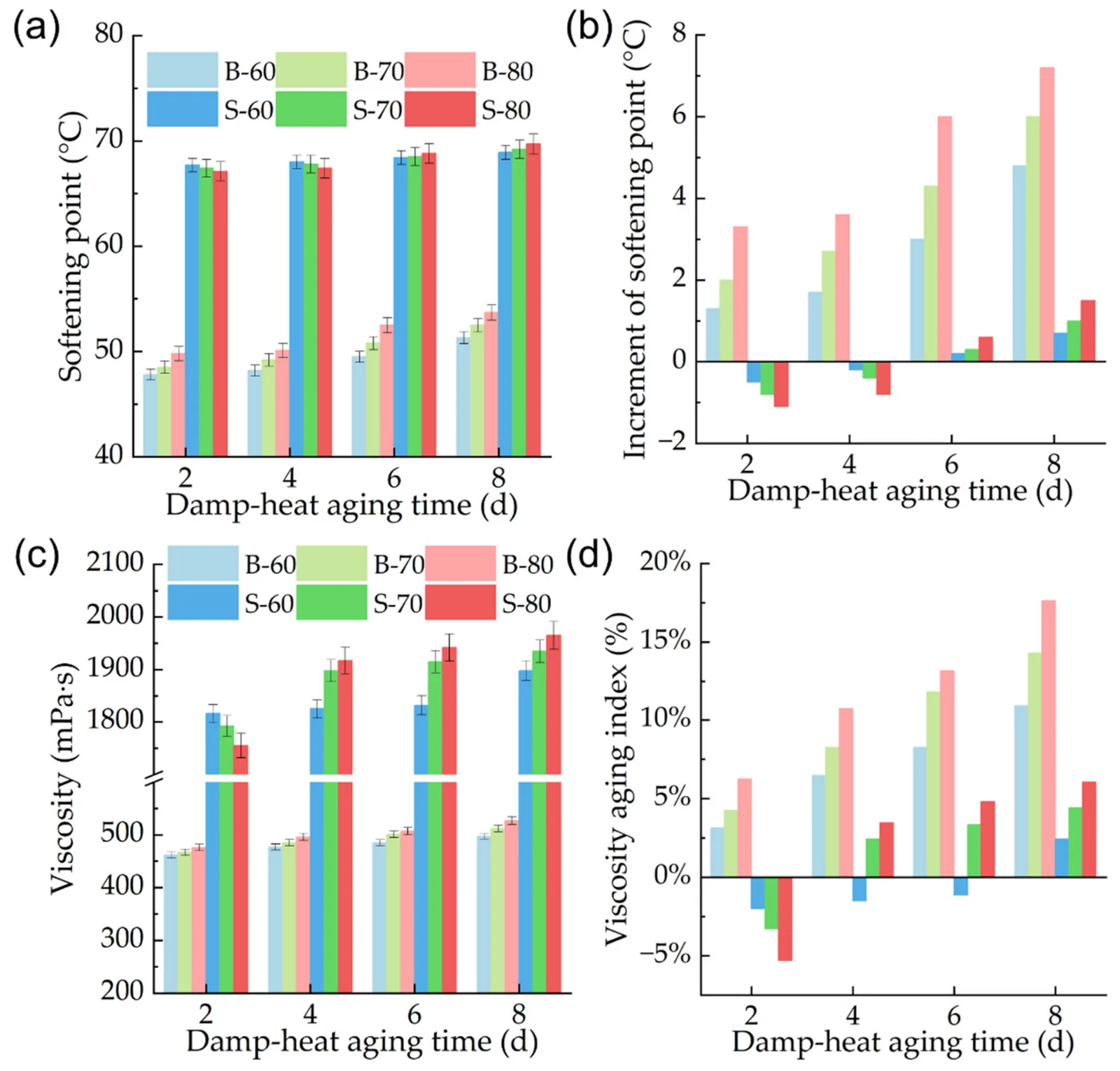
High-temperature performance of asphalt binder under various damp-heat aging conditions: (**a**) softening point; (**b**) increasement of softening point; (**c**) viscosity; (**d**) viscosity aging index.

The softening points and Brookfield viscosities of different asphalt binders are presented in Figure 6. As shown in Figure 6a,c, the base asphalt exhibited typical monotonic hardening behavior, with both softening point and viscosity increasing as the damp-heat aging time and temperature rose. For instance, the B-80 sample experienced an increase in softening point from 46.5 °C to 53.7 °C and a sharp rise in viscosity from 448 mPa·s to 527 mPa·s after 8 days’ aging. The corresponding softening point increment and viscosity aging indices are illustrated in Figure 6b,d. After 8 days, the B-80 sample reached an increased softening point (ISP) of 7.2 °C and a viscosity aging index (VAI) of 17.6%, with the increases in both indices at high temperature notably exceeding those at 60 °C. This behavior is primarily attributed to the enhanced molecular thermal motion of base asphalt at elevated temperatures, which, under moisture exposure, facilitates hydrolysis and ionization of light components, thereby promoting asphalt hardening.

From the perspective of macroscopic properties (softening point and viscosity), the damp-heat aging of base asphalt proceeds relatively uniformly. In contrast, SBS-modified asphalt undergoes a more complex, two-stage aging process. During the initial aging period (2–4 days), particularly at higher temperatures, both the softening point and viscosity of SBS asphalt exhibited a temporary decline. The softening point of the S-80 sample decreased from 68.2 °C to 65.5 °C, while its viscosity dropped from 1853 mPa·s to 1755 mPa·s. The corresponding ISP and VAI also showed negative values or minimal increases, with the early-stage VAI of S-80 reaching −5.3%. This anomalous behavior is likely due to the solubilization of water in SBS asphalt during the initial stage of damp-heat aging, with minimal loss of light components. Additionally, residual moisture within the SBS asphalt may contribute to the transient reduction in softening point and viscosity. Furthermore, the surface SBS molecular chains may undergo partial hydrolysis and chain scission under high-temperature and high-humidity conditions, partially compromising the three-dimensional elastic network and temporarily weakening its reinforcement effect on the asphalt matrix. As aging progresses into the second stage (4–8 days), the performance of SBS asphalt recovers rapidly, with the magnitude of change increasing at higher temperatures. The S-80 sample ultimately reached a viscosity of 1965 mPa·s, representing increases of 6.0% compared to the unaged sample. This stage is dominated by oxidative hardening of the asphalt, leading to enhanced high-temperature performance.

#### 3.2.2. Effects of Damp-Heat Aging on the Low-Temperature Performance of SBS-Modified Asphalt

**Figure 7:**
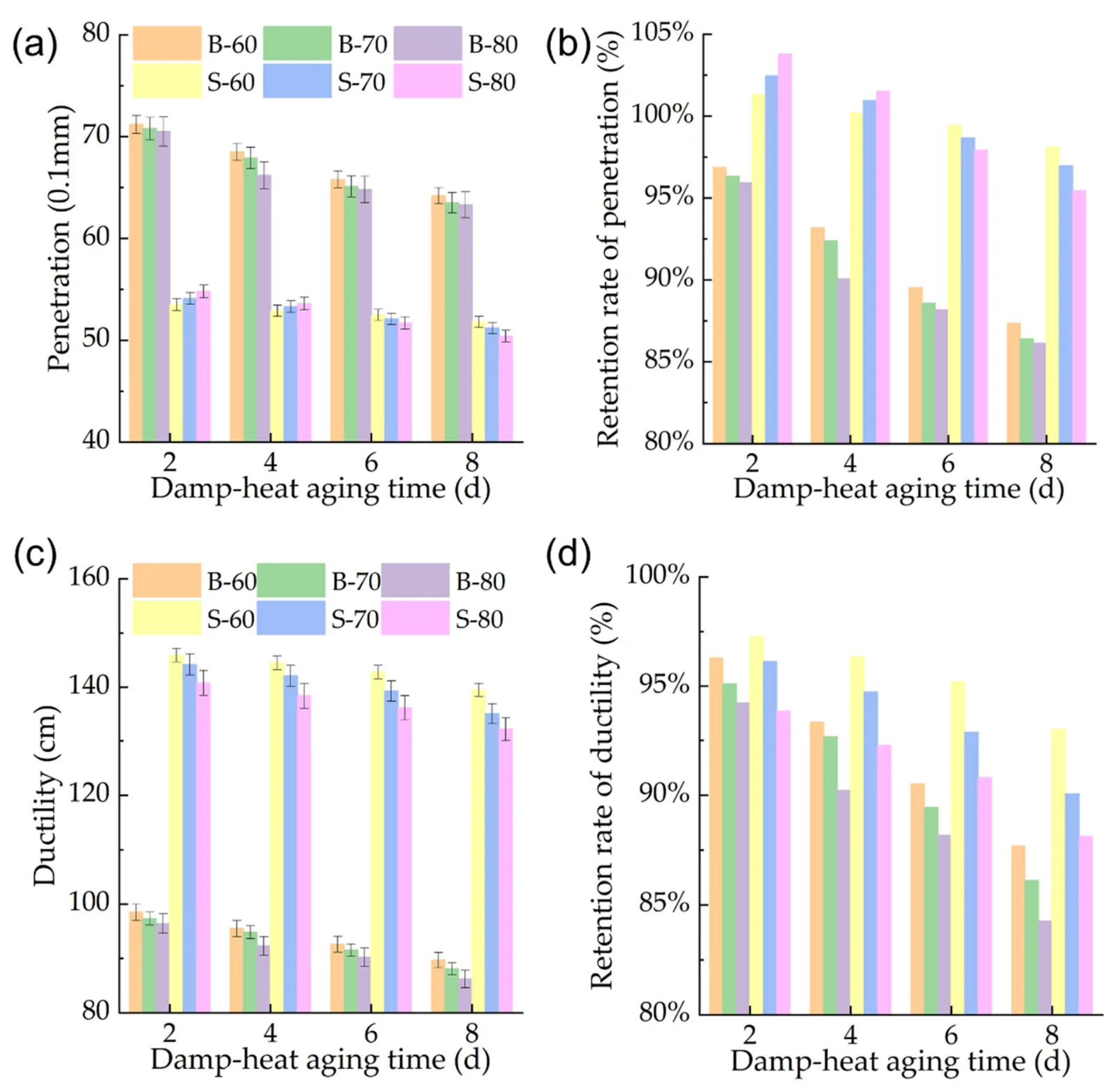
Low-temperature performance of asphalt binder under various damp-heat aging conditions: (**a**) penetration; (**b**) retention rate of penetration; (**c**) ductility; (**d**) retention rate of ductility.

Figure 7 compares the effects of damp-heat aging on the penetration, ductility, and their retention rates (RRP and RRD) of base asphalt and SBS-modified asphalt. The penetration of base asphalt decreased continuously with damp-heat aging; for instance, the B-80-8 sample dropped from 73.5 to 63.3, corresponding to a penetration retention rate (RRP) reduction of 13.9%, indicating that high temperatures exacerbate damp-heat aging. Ductility exhibited a similar linear decline, with B-80-8 decreasing from 102.3 cm to 86.2 cm. In contrast, SBS-modified asphalt demonstrated a distinctive non-monotonic behavior. During the early aging stage (0–2 days), penetration temporarily increased rather than decreased, with the S-80 sample rising from 52.8 to 54.8, yielding an RRP of 103.8%, likely due to a transient softening effect induced by moisture ingress. As aging progressed (4–8 days), the penetration of S-80 gradually decreased to 50.4, and RRP declined to 95.5%, remaining higher than that of the contemporaneous base asphalt (86.1%). Although ductility decreased overall, the reduction in RRD for SBS asphalt was smaller than that of base asphalt, indicating that temperature exerts a more pronounced negative impact on the ductility retention of base asphalt.

### 3.3. Effects of Damp-Heat Aging on the High-Temperature Rheological Properties of SBS-Modified Asphalt

**Figure 8:**
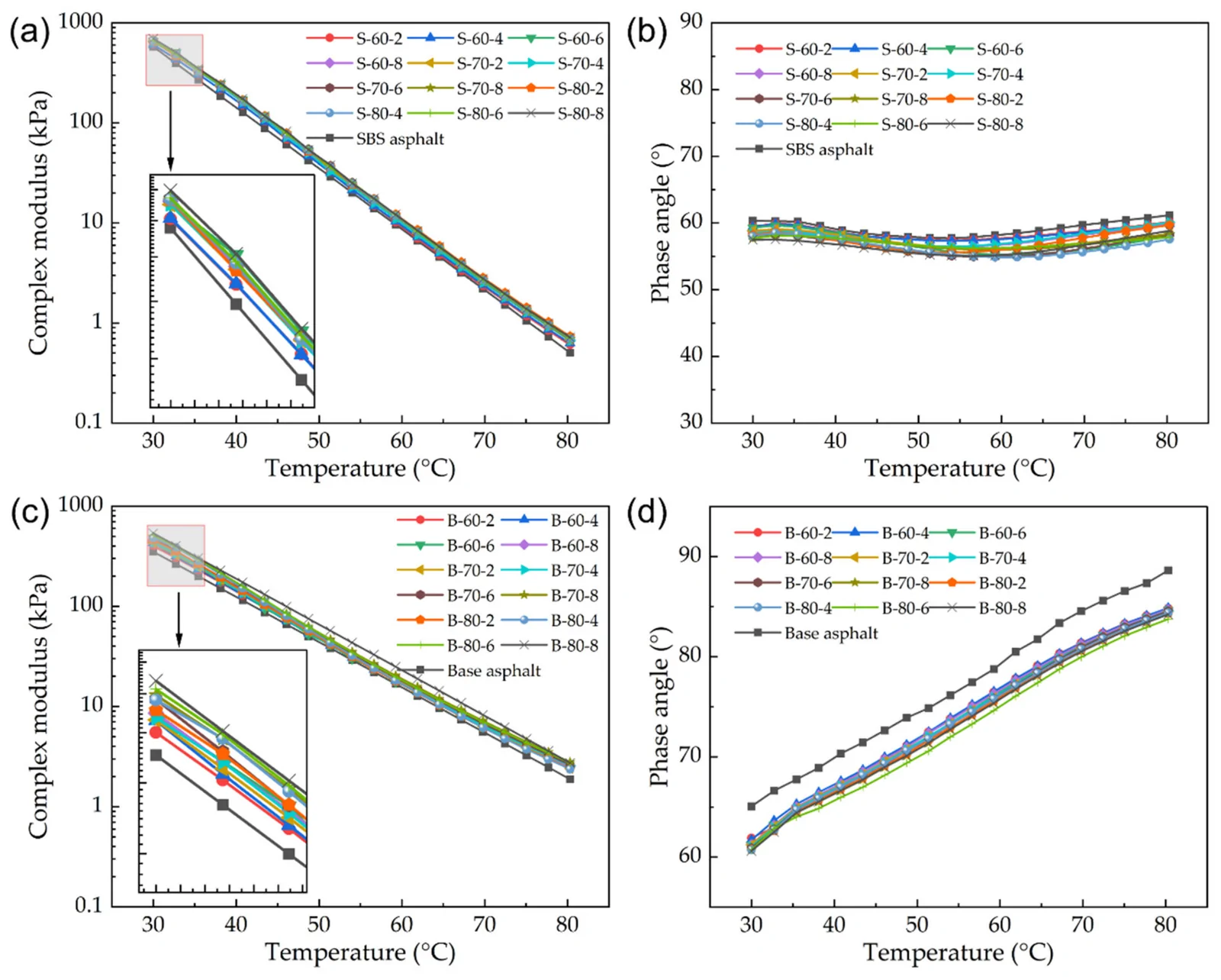
High-temperature rheological property: complex modulus of (**a**) SBS asphalt, (**c**) base asphalt; phase angle of (**b**) SBS asphalt, (**d**) base asphalt.

Figure 8 illustrates the effects of damp-heat aging on the high-temperature rheological properties of asphalt binders. Figure 8a,c present the modulus characteristics of SBS-modified asphalt and base asphalt under different damp-heat aging conditions. As the aging time and temperature increased, the initial complex modulus of both asphalt types increased, although the magnitude of change differed significantly. For example, at an initial temperature of 29.99 °C, the unaged SBS-modified asphalt exhibited a modulus of 578.72 MPa, which increased to 697.89 MPa after 8 days at 80 °C, representing a 20.59% increase. In contrast, the base asphalt increased from 351.74 MPa to 538.49 MPa under the same conditions, corresponding to a 53.09% increase. This indicates that damp-heat aging noticeably enhances asphalt stiffness, with base asphalt being more sensitive to aging. Figure 8b,d show the phase angle characteristics of SBS and base asphalt, reflecting the viscoelastic behavior of the samples. With increasing aging, the phase angle of both asphalt types decreased, indicating hardening due to aging. It was observed that asphalt aged for 4 days at 80 °C exhibited viscoelastic characteristics similar to those aged for 8 days at 70 °C. These results demonstrate that higher aging temperatures substantially accelerate binder aging for both SBS-modified and base asphalt.

**Figure 9:**
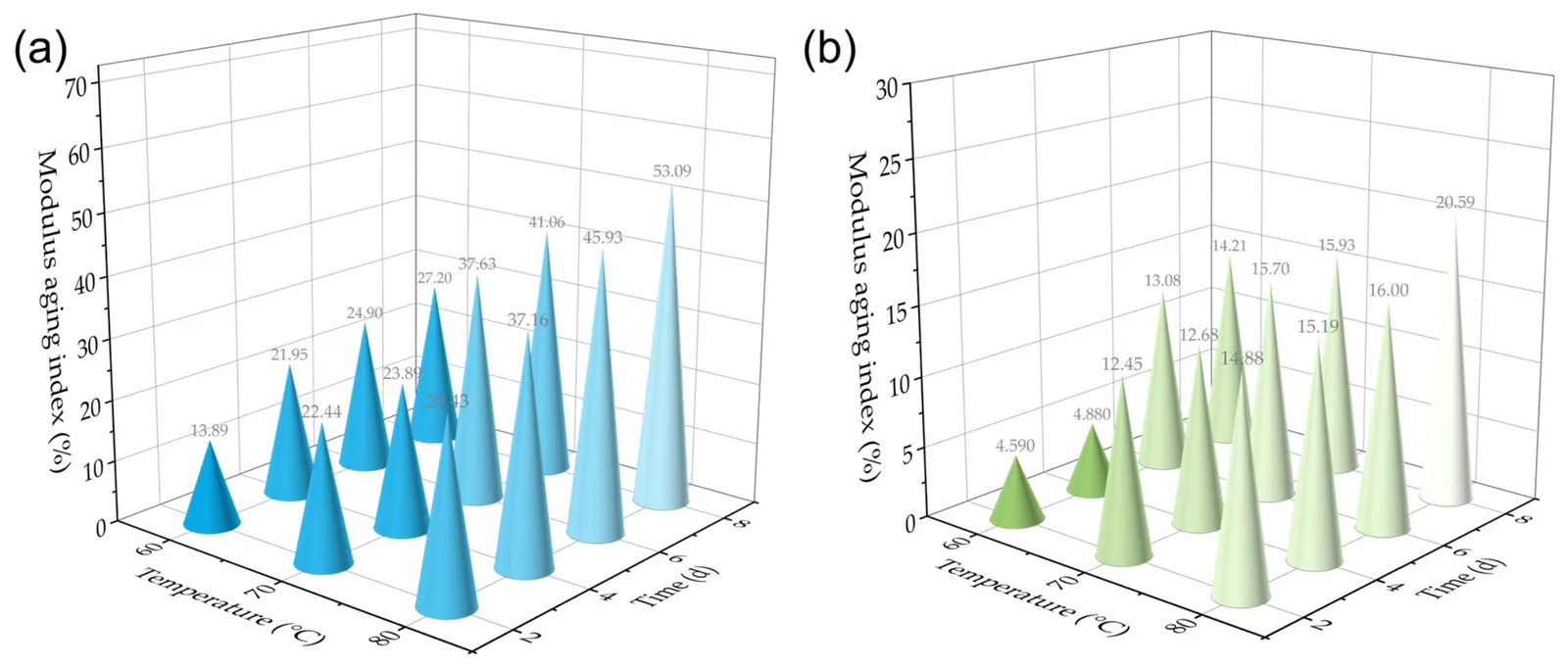
Modulus aging index: (**a**) base asphalt; (**b**) SBS-modified asphalt.

The modulus aging index (MAI), calculated based on the initial modulus at 29.9 °C, further quantifies the impact of damp-heat aging, as shown in Figure 9. Figure 9a,b present the MAI for base asphalt and SBS-modified asphalt, respectively. The MAI increased with both aging temperature and duration, with base asphalt exhibiting substantially higher values than SBS-modified asphalt. After 8 days at 80 °C, the MAI of base asphalt reached 53.09%, whereas that of SBS-modified asphalt was only 20.59%. Notably, temperature exerted a much stronger influence on aging than time. When the aging temperature increased from 60 °C to 80 °C, the MAI of base asphalt rose by nearly 50%, while that of SBS-modified asphalt increased by only approximately 30%. These results indicate that SBS-modified asphalt exhibits superior resistance to damp-heat aging at elevated temperatures.

### 3.4. Effects of Damp-Heat Aging on the Low-Temperature Rheological Properties of SBS-Modified Asphalt

**Figure 10:**
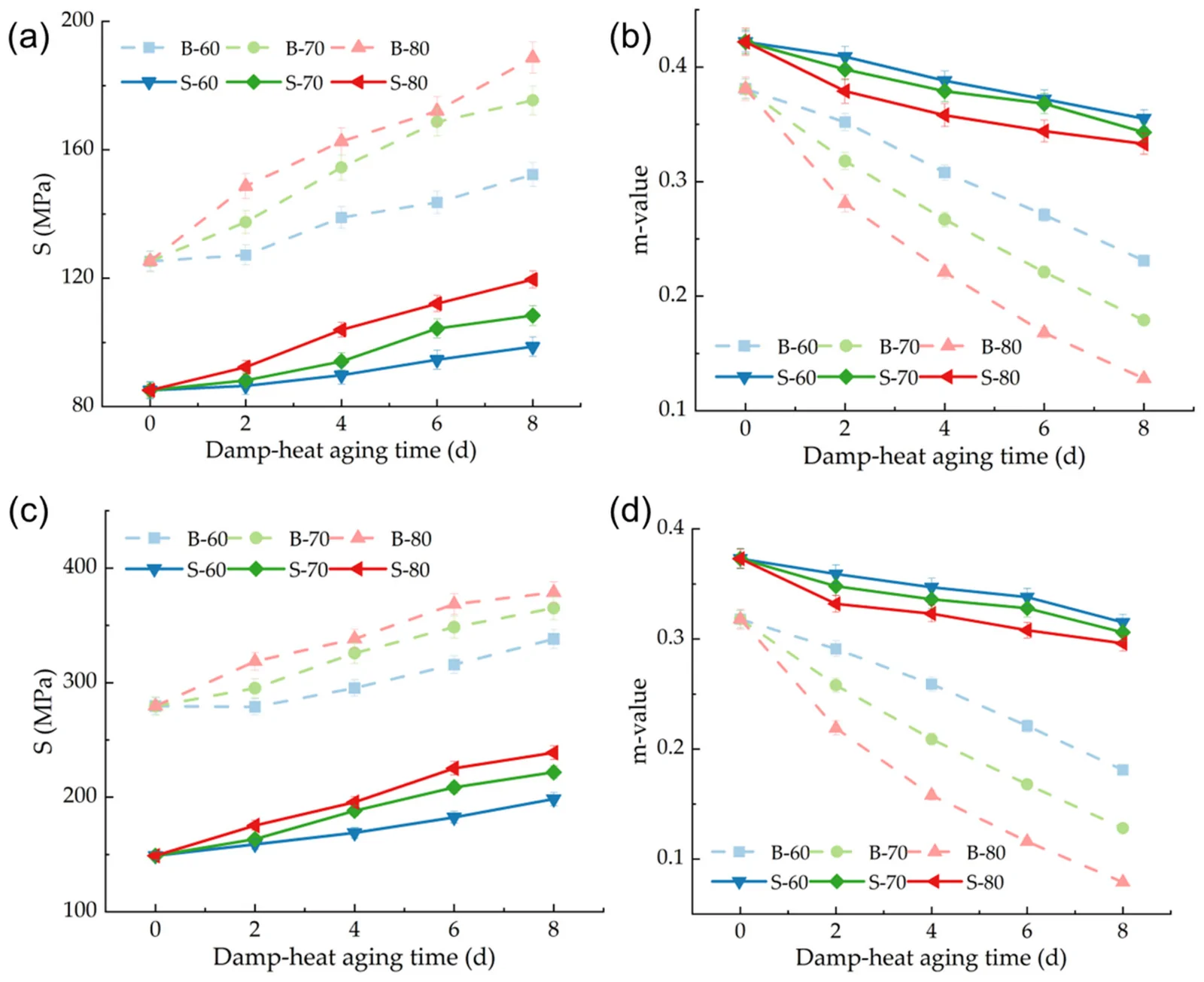
Low-temperature rheological property: (**a**) creep stiffness at −12 °C; (**b**) creep rate at −12 °C; (**c**) creep stiffness at −18 °C; (**d**) creep rate at −18 °C.

Figure 10 illustrates the effects of damp-heat aging on the rheological properties of asphalt binders at −12 °C (Figure 10a,b) and −18 °C (Figure 10c,d). After 8 days of aging at 80 °C, the base asphalt (B-80) exhibited a dramatic increase in creep stiffness (S) from 125.3 MPa to 188.7 MPa (a 50.6% increase) and a decrease in creep rate (m-value) from 0.381 to 0.128 (a 66.4% reduction), indicating a significant increase in brittleness and severe deterioration of stress relaxation capacity. In contrast, SBS-modified asphalt (S-80) under the same conditions showed an increase in S from 85.1 MPa to 119.6 MPa (a 40.5% rise) and a decrease in m-value from 0.422 to 0.333 (a 21.1% reduction), which remains above the specification threshold of 0.3, demonstrating a notably smaller degree of performance degradation. This comparison suggests that the SBS polymer network may help delay the hardening and embrittlement induced by aging. Notably, aging temperature has a far greater effect on performance deterioration than aging duration. For example, the m-value of base asphalt aged at 80 °C for 2 days (0.281) was already lower than that aged at 60 °C for 4 days (0.308), confirming that the synergistic effect of high temperature and humidity accelerates aging damage. Although SBS-modified asphalt exhibits superior aging resistance, prolonged aging brings its m value close to the critical threshold; as shown in Figure 10d, the m value of S-80-8 at −18 °C decreased to 0.296.

### 3.5. Effects of Damp-Heat Aging on the Chemical Functional Groups of SBS-Modified Asphalt

**Figure 11:**
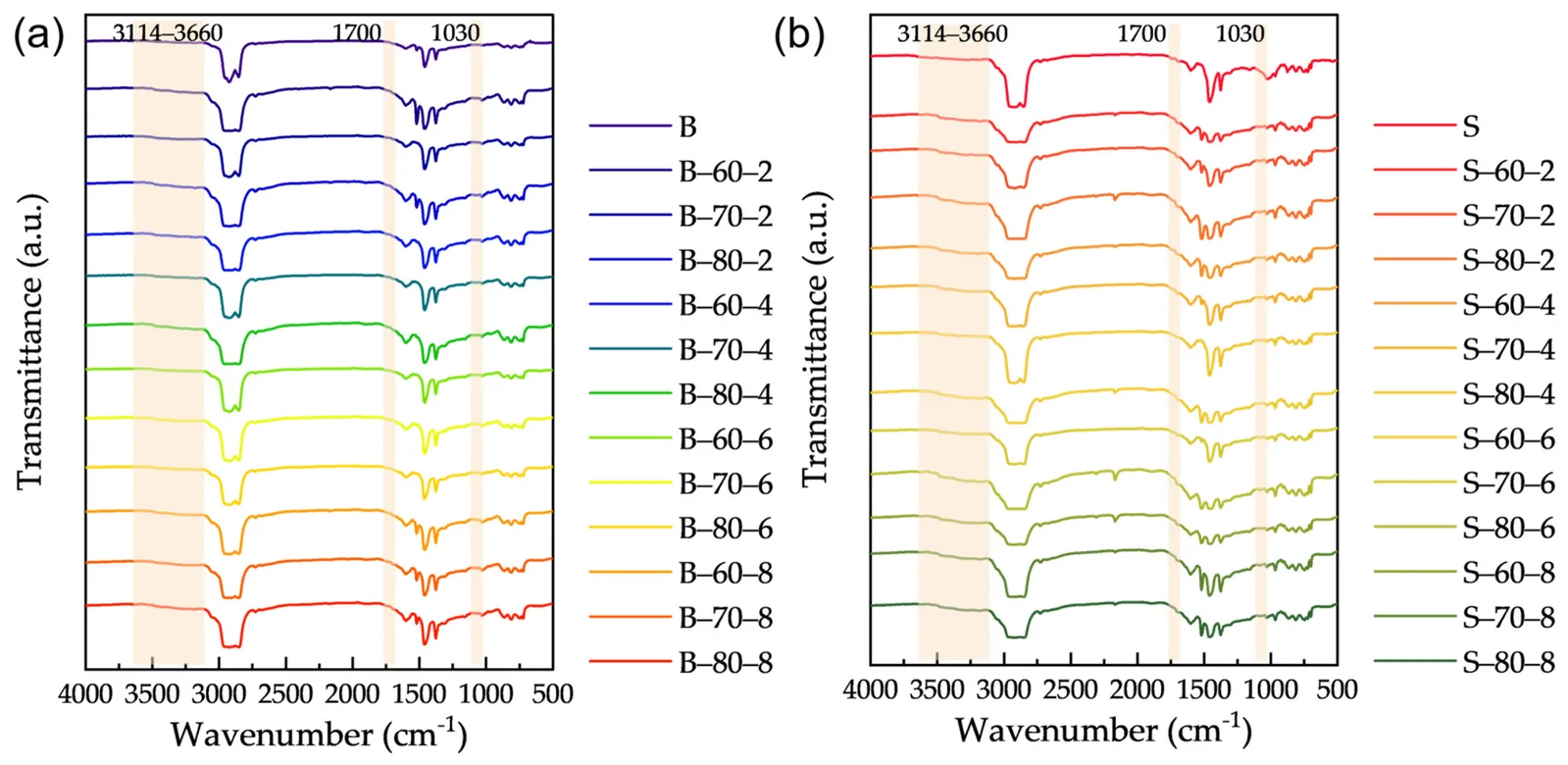
Infrared spectrum of asphalt binder under various damp-heat aging conditions: (**a**) base asphalt; (**b**) SBS asphalt.

Figure 11 presents the FTIR spectra of asphalt samples after different aging durations. During damp-heat aging, changes in the chemical structure of asphalt can be quantitatively characterized by the variation in functional group indices. Based on the infrared data, the relative contents of three functional groups associated with damp-heat aging—sulfoxide (S=O), carbonyl (C=O), and hydroxyl (O–H)—were calculated, and the results are shown in Figure 11. As the aging temperature and duration increased, all three functional group indices of both asphalt types exhibited an upward trend, although the magnitude and pattern of change differed significantly. For base asphalt (B-80-8), the sulfoxide index increased from 0.00458 to 0.00776, a rise of approximately 69.2%, while the carbonyl and hydroxyl indices increased by about 55.3% and 185.3%, respectively. In contrast, the corresponding increases for SBS-modified asphalt (S-80-8) were approximately 114.7%, 97.6%, and 54.8%. These results indicate that oxidative aging is more severe in base asphalt, whereas the presence of the SBS polymer network partially delays the oxidation process. However, the pronounced increase in sulfoxide suggests that the C=C bonds in the SBS polymer may be more susceptible to oxidation under damp-heat conditions. Notably, the hydroxyl index of B-80-2 exhibited the most significant rise in the early aging stage, reaching 46.0% before leveling off, which is closely related to the saturation process of moisture ingress.

**Figure 12:**
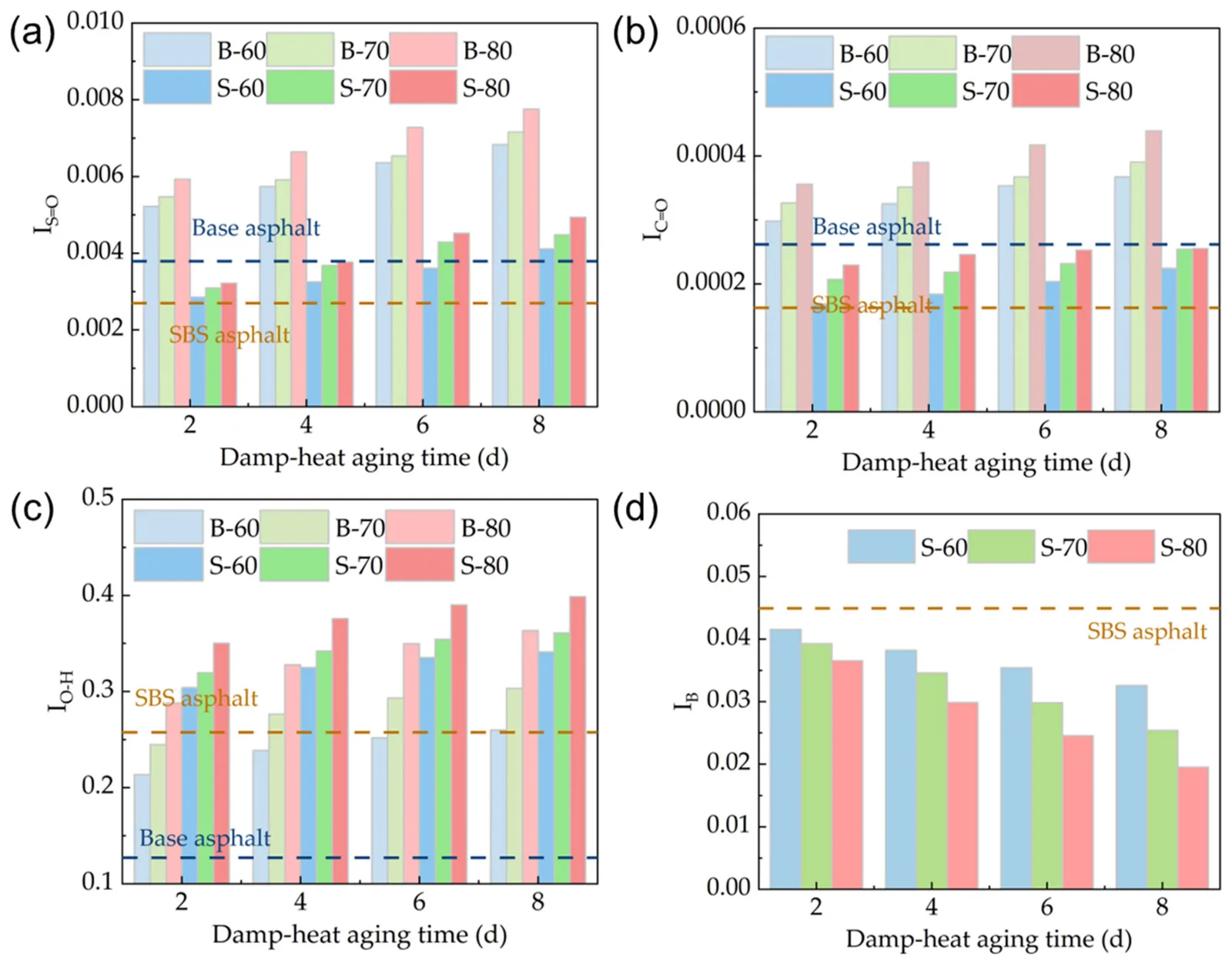
Aging index of chemical functional group of asphalt binder under various damp-heat aging conditions: (**a**) sulfoxide index; (**b**) carbonyl index; (**c**) hydroxyl index; (**d**) butadiene index.

The distinctive feature of damp-heat aging lies in the synergistic effect of moisture and thermal oxidation. In the early stage of aging, as shown in Figure 12, the hydroxyl index of B-80-2 sharply increased from 0.12738 to 0.28812, likely due to rapid penetration and adsorption of moisture. This process temporarily disrupts intermolecular interactions within the asphalt, resulting in a transient decrease in the macroscopic softening point. As aging progresses (2–8 days), thermal-oxidative reactions gradually dominate, with the sulfoxide and carbonyl contents of B-80 increasing continuously during this period, reflecting the formation of asphalt aging products that induce hardening. In contrast, the changes in functional group indices for SBS-modified asphalt are generally smaller, might because the SBS polymer network slows moisture penetration and preferentially undergoes oxidation, thereby protecting the asphalt matrix. Therefore, damp-heat aging of asphalt represents a process evolving from moisture-dominated initial plasticization to thermally and oxidatively driven long-term hardening. The monotonic increase in sulfoxide and carbonyl indices serves as an indicator of oxidation severity, whereas abrupt changes in the hydroxyl index reveal the critical window of moisture ingress.

## 4. Effect of Damp-Heat Aging on Steel Slag Asphalt Mixture

### 4.1. Microscopic Morphology of Steel Slag Aggregates and Their Adhesion to Asphalt

#### 4.1.1. Surface Morphology

**Figure 13:**
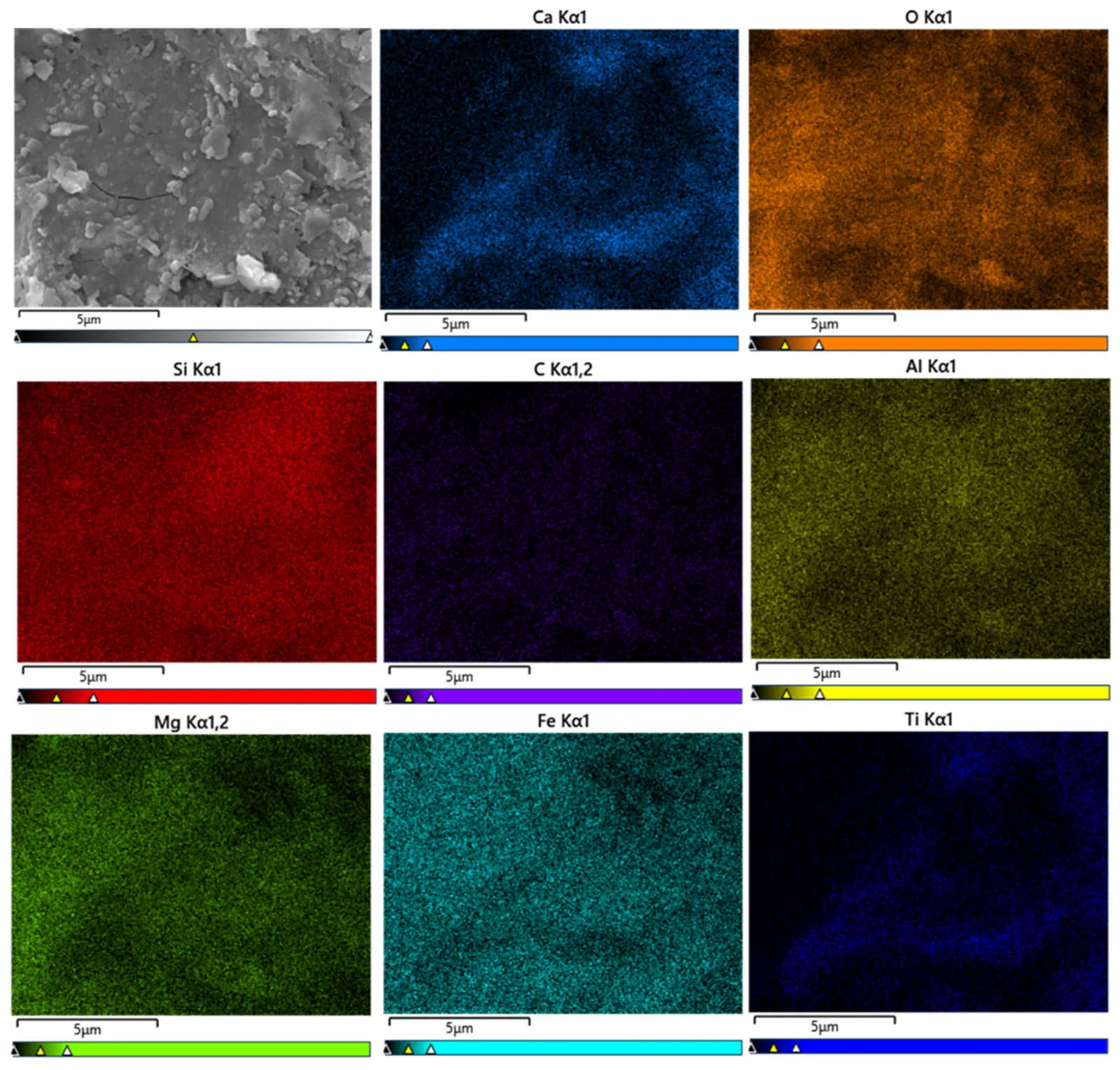
Surface morphology and elemental distribution of basalt aggregates.

**Figure 14:**
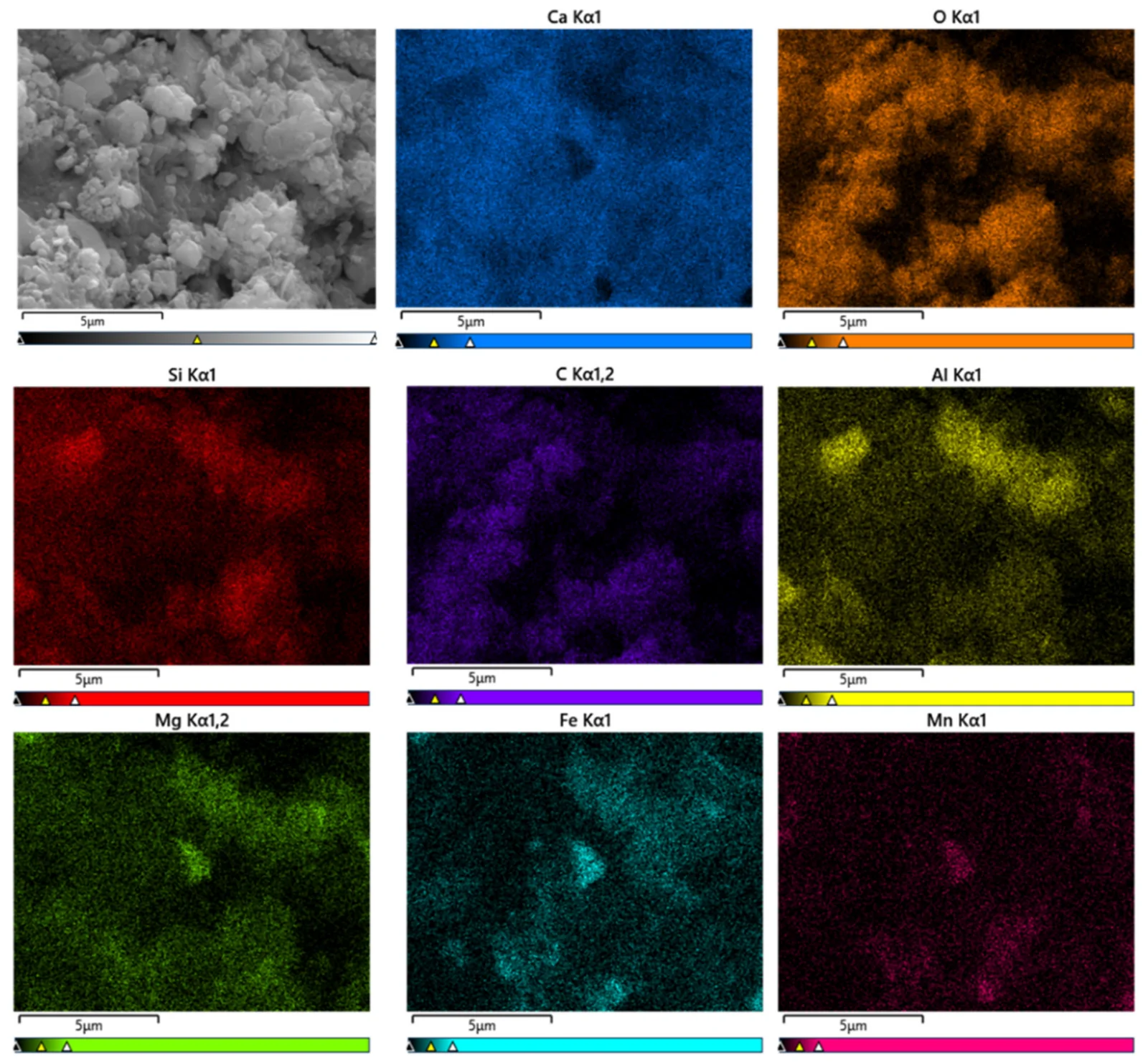
Surface morphology and elemental distribution of steel slag.

The surface micro-texture and chemical mineral composition of aggregates are critical determinants of the adhesion quality at the asphalt-aggregate interface, particularly under damp-heat conditions. The combined SEM and EDS analyses of the steel slag and basalt aggregates, illustrated in Figure 13 and Figure 14 and Table 10 and Table 11, reveal substantial differences in their physicochemical properties. As depicted in Figure 13 and Table 10, the basalt surface exhibits smooth and dense fracture morphologies and is highly enriched in silicon (Si content reaching 14.98%). This characteristic acidic silicate surface is strongly hydrophilic, making it highly prone to water molecule adsorption in damp-heat environments. Consequently, this can establish a percolation pathway for moisture intrusion, ultimately leading to the physical stripping of the asphalt film.

In contrast, Figure 14 reveals that the surface of the steel slag aggregate is densely populated with micrometer-scale pores formed by gas evolution during the smelting process. This rough, porous micro-texture not only provides a noticeably larger specific surface area for asphalt contact but also facilitates a profound mechanical interlocking effect. Furthermore, as detailed in Table 11, the steel slag exhibits pronounced calcium-rich characteristics, with a Ca content reaching 28.62%. The alkaline constituents on the steel slag surface are hypothesized to exert a critical chemical anchoring effect at the aggregate-asphalt interface: based on the elemental data, these surface alkaline oxides are highly probable to undergo in-situ acid-base reactions with the acidic functional groups (e.g., carboxylic acids and anhydrides) generated within the thermo-oxidatively aged asphalt, potentially yielding water-insoluble, chemically bonded products. Therefore, distinct from the singular and relatively weak physical adhesion observed in basalt, steel slag fundamentally alters the interfacial resistance to moisture damage through the dual synergistic effect of physical-mechanical interlocking and chemical anchoring. This synergy effectively inhibits the competitive displacement of the asphalt film by moisture, providing a robust microstructural foundation for the mixture to sustain superior mechanical properties even following long-term damp-heat aging.

#### 4.1.2. Adhesion

**Figure 15:**
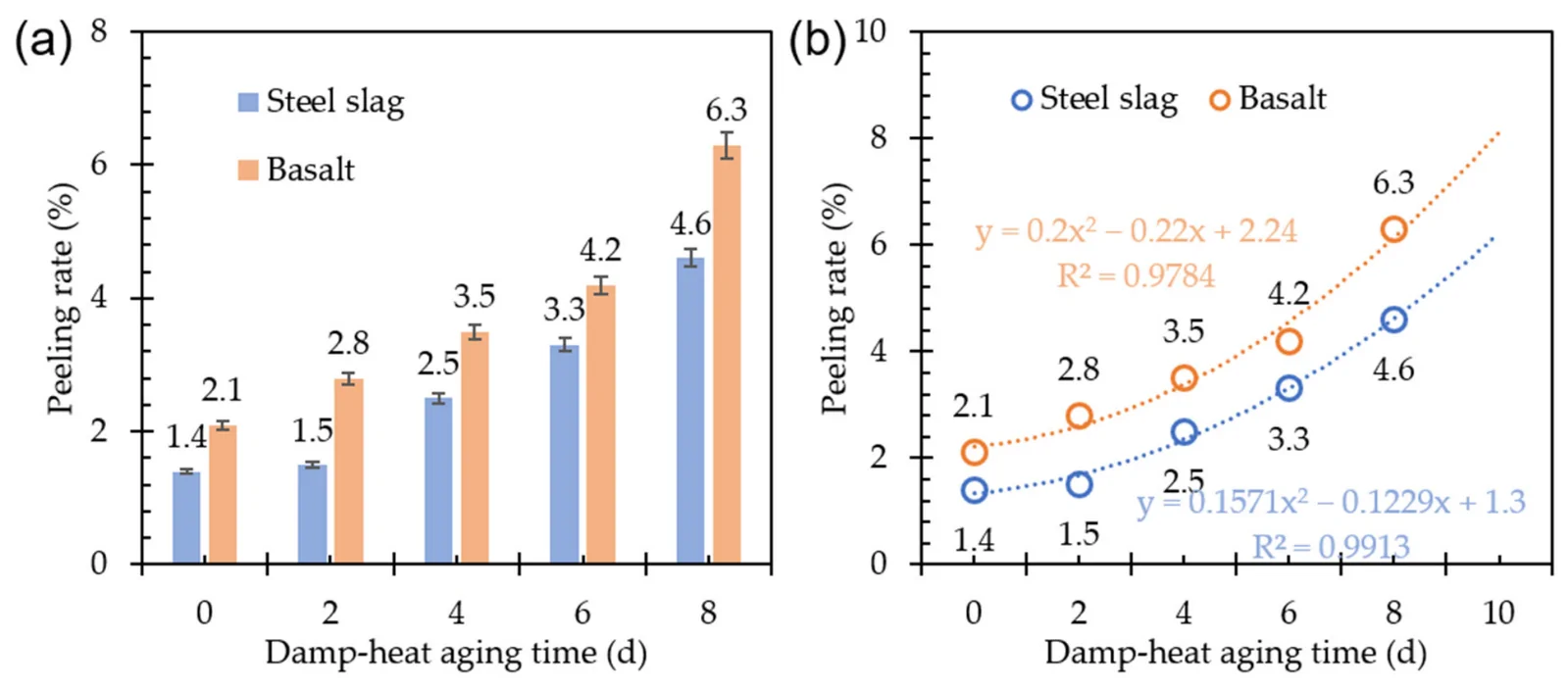
Aggregate adhesion: (**a**) stripping rate; (**b**) fitting curves.

Figure 15 illustrates the adhesion characteristics of the steel slag and basalt aggregates with asphalt subjected to damp-heat aging. For both aggregate types, asphalt stripping exacerbated as the duration of damp-heat aging increased; however, the steel slag consistently exhibited lower stripping rates across all aging stages. As depicted in Figure 15a, in the unaged state, the stripping rate of the steel slag was a mere 1.3%, compared to 2.1% for the basalt. After 8 days of aging, the stripping rate for the steel slag increased to 4.6%, which remained lower than the 6.3% observed for the basalt. Figure 15b presents the fitted curves correlating the stripping rate with the damp-heat aging time. A comparison of the quadratic coefficients from the fitting equations (0.1571 for steel slag versus 0.2000 for basalt) indicates that the stripping rate of basalt undergoes a more pronounced accelerated growth over time, signifying a higher sensitivity to aging. This performance discrepancy is primarily attributed to the rough and porous micromorphology of the steel slag aggregate, which provides superior mechanical interlocking. Furthermore, its alkaline surface characteristics are hypothesized to facilitate the formation of stable chemical adsorption with the acidic components present in the asphalt, thereby substantially enhancing interfacial adhesion. Consequently, steel slag demonstrates significant potential for application as a high-performance, anti-stripping aggregate in asphalt pavements, particularly in humid regions.

### 4.2. Road Performance of Steel Slag Asphalt Mixture Under Damp-Heat Aging

#### 4.2.1. Mechanical Performance

**Figure 16:**
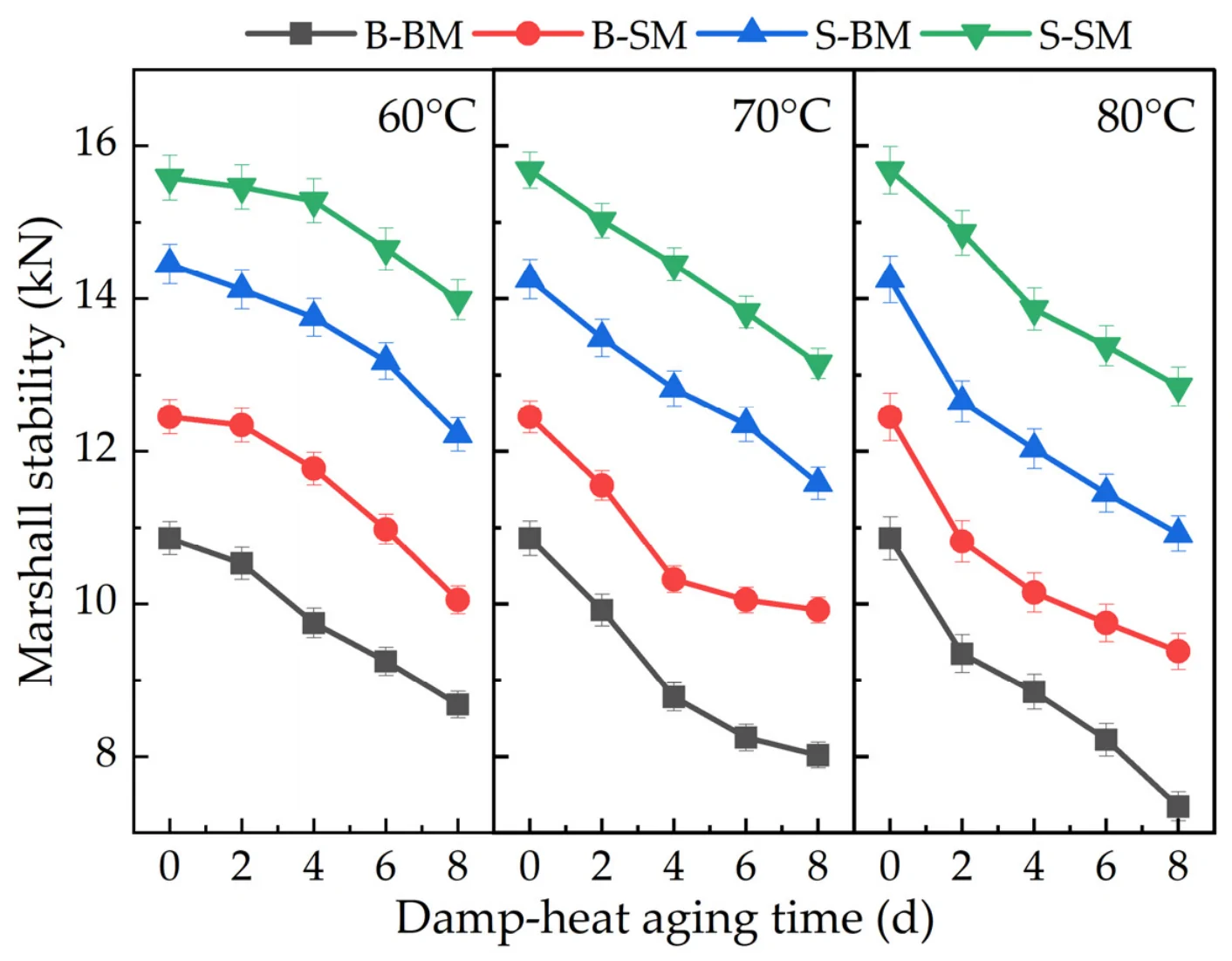
Mechanical performance of steel slag asphalt mixture under various damp-heat aging conditions.

The variation in Marshall stability (MS) serves as a direct indicator of the mixtures’ resistance to high-temperature deformation during the damp-heat aging process. As illustrated in Figure 16, although the stability of all mixtures exhibited a declining trend as aging conditions intensified, the steel slag aggregates demonstrated a skeletal support capacity superior to that of basalt. Under identical aging conditions, the residual stability of the steel slag groups consistently surpassed that of the basalt groups. For instance, utilizing SBS modified asphalt, the MS value of the steel slag mixture (S-SM) was maintained at 12.85 kN following severe aging at 80 °C for 8 days. This is 14.5% higher than the 11.22 kN observed for the corresponding basalt mixture under identical conditions. Similarly, within the base asphalt group, the retained stability of the steel slag mixture (B-SM, 9.68 kN) was remarkably superior to that of the basalt mixture (B-BM, 7.35 kN). This performance discrepancy is primarily attributed to the profound mechanical interlocking effect facilitated by the unique physical architecture of the steel slag aggregate. In stark contrast to the relatively smooth surface of basalt, the steel slag surface is highly angular and densely populated with micropores. During the compaction of the Marshall specimens, this rough micro-texture increases the internal friction angle between the aggregates and allows the asphalt mastic to permeate the micropores, effectively forming physical pins. Consequently, even in the advanced stages of damp-heat aging—when the cohesive strength of the asphalt binder deteriorates due to moisture damage—the robust, interlocking skeletal structure established by the steel slag continues to efficiently transfer loads and resist high-temperature-induced plastic deformation. This structural integrity ultimately guarantees the mechanical stability of the mixture in severe environments.

#### 4.2.2. Indirect Tensile Strength

**Figure 17:**
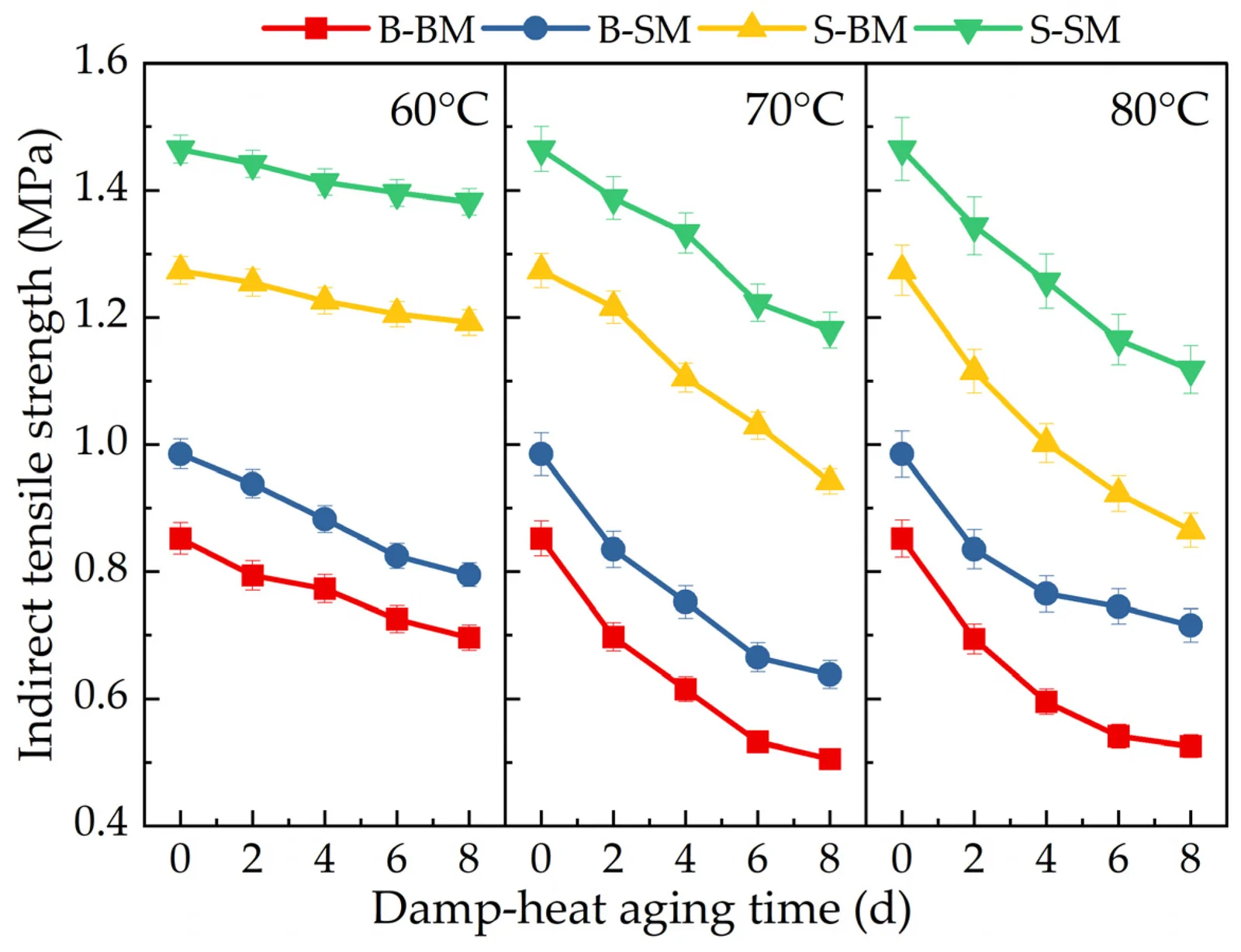
Cohesion performance of steel slag asphalt mixture under various damp-heat aging conditions.

Indirect tensile strength (ITS) serves as a highly sensitive indicator of adhesion quality at the asphalt-aggregate interface, effectively elucidating a material’s resistance to moisture damage. Figure 17 presents the ITS results for the various asphalt mixtures. While damp-heat aging induced a significant reduction in ITS across all samples, the strength degradation of the steel slag mixtures was notably decelerated, demonstrating superior resistance to moisture-induced stripping. Following aging at 80 °C for 8 days, the ITS value of the SBS-modified basalt mixture (S-BM) deteriorated to 0.865 MPa, whereas the SBS-modified steel slag mixture (S-SM) sustained a strength of 1.118 MPa, representing a remarkable 29.2% advantage. The contrast within the base asphalt group is even more pronounced: the residual ITS of the B-SM (0.715 MPa) substantially surpassed that of the B-BM (0.525 MPa), confirming that moisture-induced interfacial damage is far more severe in basalt mixtures. The primary mechanism driving this performance divergence lies in their disparate interfacial chemistries. Basalt is abundant in acidic silicon dioxide (SiO_2_), which exhibits a strong propensity to form hydrogen bonds with water molecules in damp-heat environments, inevitably leading to the physical stripping of the asphalt film. Conversely, steel slag is rich in calcium oxide (CaO), which engages in in-situ acid-base reactions with the acidic functional groups (e.g., carboxylic acids and anhydrides) generated within the asphalt during thermo-oxidative aging. These reactions yield water-insoluble chemical anchor bonds. This interfacial chemical bonding constructs a robust moisture barrier, effectively blocking the percolation and competitive displacement of water, thereby endowing the steel slag mixtures with exceptional durability against moisture damage.

#### 4.2.3. Low-Temperature Cracking Resistance

**Figure 18:**
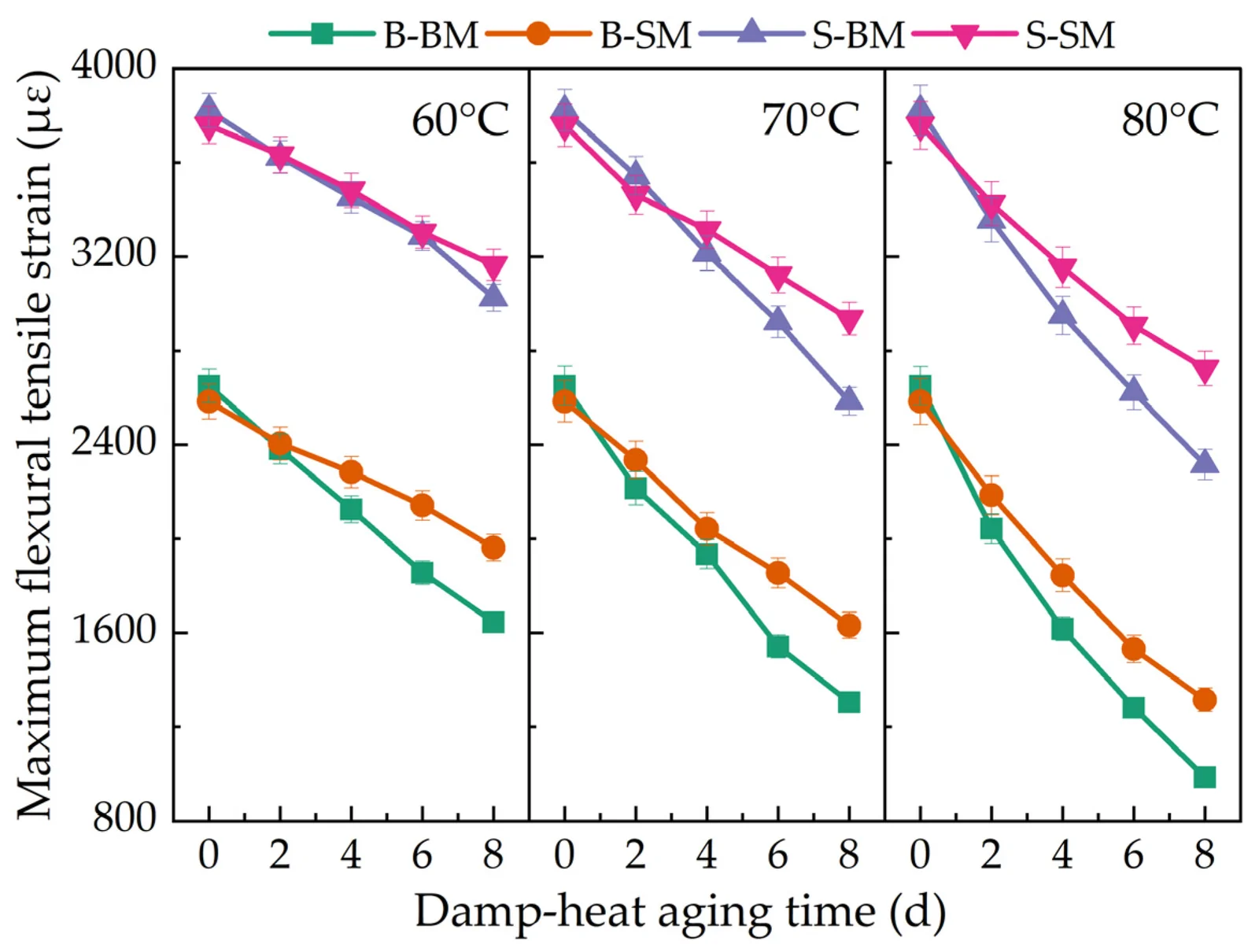
Low-temperature performance of steel slag asphalt mixture under various damp-heat aging conditions.

Damp-heat inevitably induces the hardening and embrittlement of the asphalt binder, thereby compromising the low-temperature cracking resistance of the mixture. The low-temperature flexural strain results are presented in Figure 18. Although the initial flexural strain of the steel slag mixtures in the unaged state is marginally lower than that of the basalt groups—a consequence of the inherently high modulus of the steel slag aggregate—the steel slag mixtures exhibit a superior toughness retention rate following long-term aging. Specifically, after aging at 80 °C for 8 days, the flexural strain of the SBS-modified basalt mixture (S-BM) precipitously declined to 2315 με. In contrast, the SBS-modified steel slag mixture (S-SM) sustained a robust strain level of 2725 με. Similarly, the residual strain of the base asphalt steel slag mixture (B-SM, 1315 με) noticeably exceeded that of the corresponding basalt mixture (B-BM, 986 με). These results indicate that, even in the advanced stages of aging, the steel slag mixtures avert the catastrophic brittle fracture characteristically observed in their basalt counterparts. The fundamental mechanism underlying this enhanced performance is that the robust interfacial bonding effectively suppresses both the initiation and propagation of microcracks. Under low-temperature stress conditions, the weak interfacial adhesion within the basalt mixtures allows hygrothermal-aging-induced microcracks to rapidly propagate along the aggregate-asphalt interface, ultimately culminating in premature failure. Conversely, the tight bond between the steel slag and asphalt ensures that the interfacial transition zone is no longer a mechanical weak link. Consequently, crack propagation pathways are forced to deviate either through the relatively tougher asphalt mastic matrix or directly through the aggregate itself. The preservation of this interfacial integrity ensures that the steel slag mixtures can effectively transfer stress and dissipate fracture energy even after enduring severe aging, thereby significantly prolonging the low-temperature service life of the pavement.

### 4.3. Comprehensive Analysis

**Figure 19:**
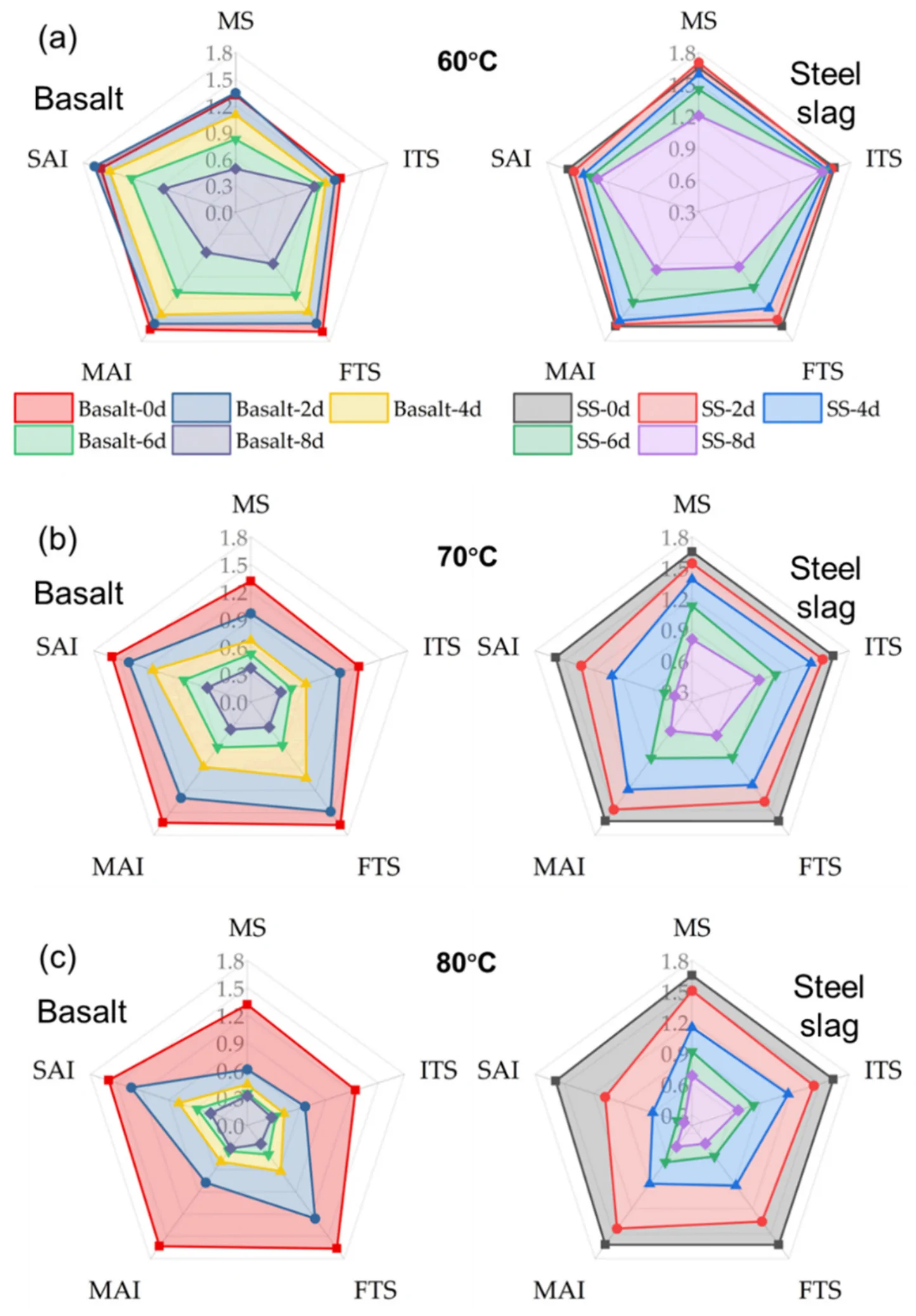
Radar diagram of steel slag asphalt mixture with different properties under wet-heat aging conditions: (**a**) 60 °C; (**b**) 70 °C; (**c**) 80 °C.

Given that SBS-modified asphalt is predominantly utilized in high-grade asphalt pavements, this section primarily compares the comprehensive performance of steel slag and basalt asphalt mixtures incorporating SBS-modified asphalt. The experimental results obtained in Section 4.2—comprising Marshall stability (MS), indirect tensile strength (ITS), low-temperature flexural strain, residual Marshall stability (RMS), and tensile strength ratio (TSR)—were subjected to standardization and nonlinear transformation to derive comprehensive evaluation indices. These multidimensional indices are visually represented in radar charts (Figure 19). As the degree of aging intensified, the enclosed area of the radar chart for the B-BM group (base asphalt with basalt) contracted rapidly. Specifically, pronounced indentations emerged along the axes corresponding to moisture damage resistance (TSR) and low-temperature performance, revealing severe performance vulnerabilities. In stark contrast, the S-SM group (SBS-modified asphalt with steel slag) consistently maintained the largest coverage area and the most geometrically balanced shape throughout the aging stages.

**Figure 20:**
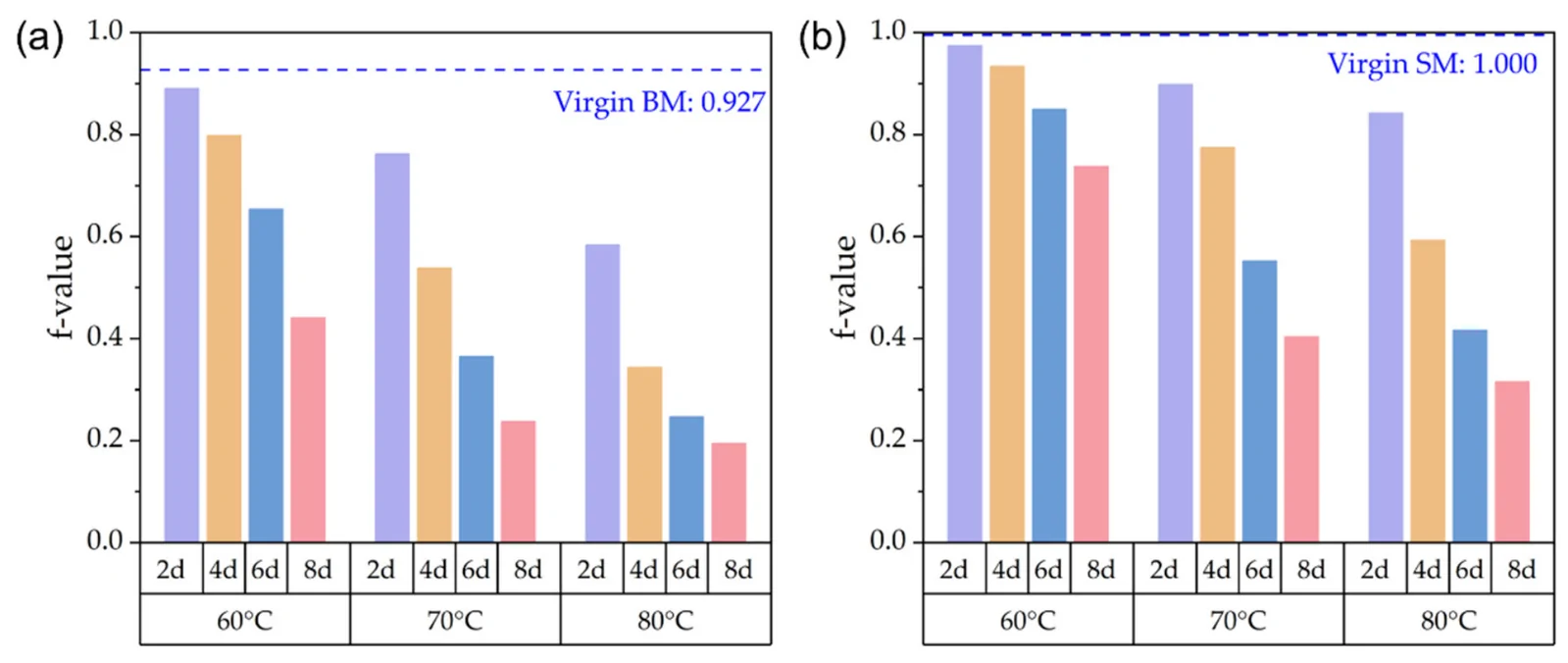
The comprehensive performance evaluation indicator (f-value): (**a**) Basalt asphalt mixture; (**b**) Steel slag asphalt mixture.

Figure 20 shows the comprehensive evaluation index, f-value, derived from the modified radar chart method. The f-value facilitates the normalized quantitative characterization of the full-lifecycle service performance of asphalt mixtures under coupled damp-heat conditions. The trajectory of performance evolution distinctly manifests the characteristics of the Time–Temperature Superposition (TTS) principle. Specifically, elevated temperatures highly accelerate the kinetic rates of moisture diffusion and oxidative reactions. Consequently, short-term high-temperature aging induces cumulative damage equivalent to, or even exceeding, that of long-term low-temperature aging (e.g., the f-value of the S-BM group at 80 °C/4 d was 0.3434, which is notably lower than the 0.4407 recorded at 60 °C/8 d). In the unaged state, the SBS-modified steel slag mixture (S-SM) achieved an f-value of 1.0, outperforming the corresponding basalt group (S-BM, 0.9270). As the time–temperature equivalent increased, the f-value of the S-BM group precipitously collapsed to 0.1949 under the extreme aging conditions of 80 °C/8 d. This represents a staggering decay rate of 79.0%, signifying that the material was approaching structural failure. In stark contrast, the S-SM group—benefiting from the profound physical and chemical anchoring at the steel slag interface—effectively retarded the performance degradation induced by the time–temperature superposition. Its residual f-value was sustained at 0.3151 (a lower decay rate of 68.5%). These findings substantiate that steel slag aggregates not only elevate the upper limits of the material’s initial performance but also substantially diminish the mixture’s time–temperature sensitivity to damp-heat environments, thereby exhibiting exceptional long-term durability.

**Figure 21:**
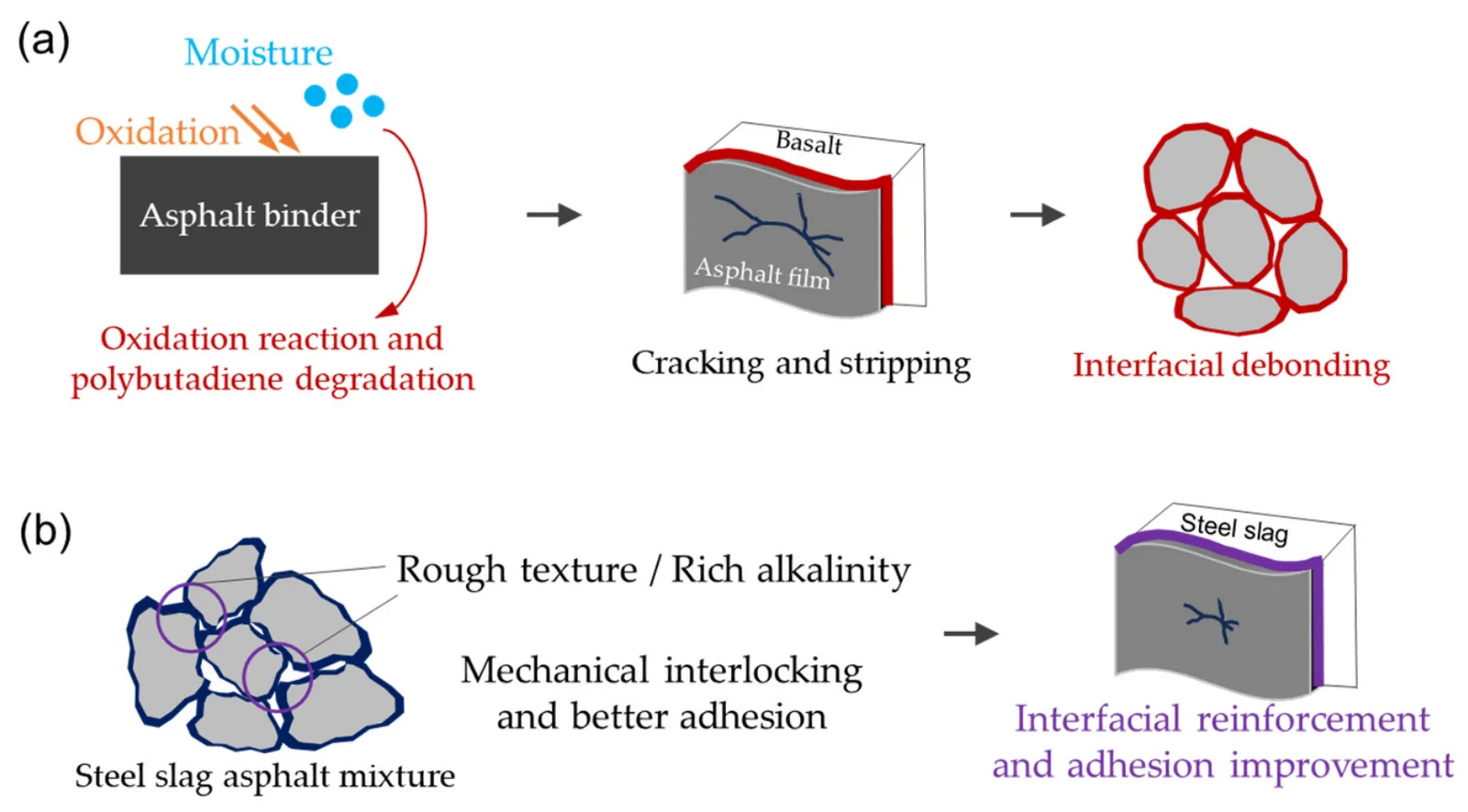
Damp-heat aging erosion mechanism of asphalt mixture interface: (**a**) Interfacial debonding of basalt asphalt mixture; (**b**) Interfacial reinforcement and adhesion improvement of steel slag asphalt mixture.

This substantial enhancement in comprehensive performance fundamentally originates from the interfacial and skeletal reinforcement mechanisms imparted by the steel slag, as summarized in Figure 21. As illustrated in Figure 21a, environmental factors such as moisture, oxygen, and heat within the damp-heat environment progressively erode the asphalt film. This prolonged exposure leads to the depletion of light components within the asphalt binder, thereby inducing severe hardening and embrittlement. Given the inherent hydrophilicity of basalt, moisture that diffuses and permeates through the asphalt film readily migrates along the asphalt-aggregate interface. Exacerbated by the continuous expansion of the moisture-eroded area and the application of external mechanical loads, the basalt asphalt mixture undergoes progressive cracking and physical stripping of the asphalt film. This degradation severely compromises the internal interfacial strength of the mixture, rapidly culminating in macroscopic pavement distresses such as potholes and raveling.

Steel slag aggregates impart a dual physical-chemical enhancement mechanism to the asphalt mixture, as illustrated in Figure 21b. During its formation process, the evolution of gases generates an abundance of micropores and a highly rough surface micro-texture on the steel slag. During the mixing and compaction phases, the hot asphalt binder permeates these microscopic voids. Upon cooling, it solidifies to form a profound mechanical interlocking structure, analogous to countless microscopic pins. This physical anchoring effect enhances the asphalt coating ratio of the steel slag aggregate, effectively suppressing moisture intrusion at the binder-aggregate interface and elevating the interfacial shear resistance. Furthermore, the pronounced angularity and superior intrinsic mechanical properties of the steel slag aggregates provide an increased number of skeletal contact points, thereby fortifying the mixture against external mechanical loading. Chemically, the abundant alkaline constituents within the steel slag are highly probable to react with the acidic functional groups (e.g., -COOH, C=O) present in the asphalt to form robust chemical bonds [34]. These hypothesized bonds possess a binding strength substantially higher than mere physical adsorption, thereby effectively resisting competitive displacement by water molecules. Consequently, the steel slag asphalt mixture achieves a profound synergistic enhancement through the combination of mechanical interlocking within the micro-pores and chemical anchoring via interfacial acid-base reactions. This powerful synergy ensures that the mixture retains exceptional structural integrity and durability even after enduring severe damp-heat aging.

### 4.4. Engineering Application

To directly validate the long-term field performance and durability of the steel slag asphalt mixture in a realistic damp-heat environment, a full-scale field test section was constructed and continuously monitored for two years in Shenzhen, Guangdong Province, a region characteristic of China’s typical hot and humid climates. The steel slag and basalt asphalt pavements were both constructed as 300 m long test sections with a width of 7.5 m and a thickness of 0.04 m, located on a municipal road in Shenzhen, China. By 2025, the annual traffic volume reached 2633 thousand vehicles, including 483 thousand heavy vehicles. During the service period, the maximum ambient temperature was approximately 32 °C, and the relative humidity exceeded 80% RH.

**Figure 22:**
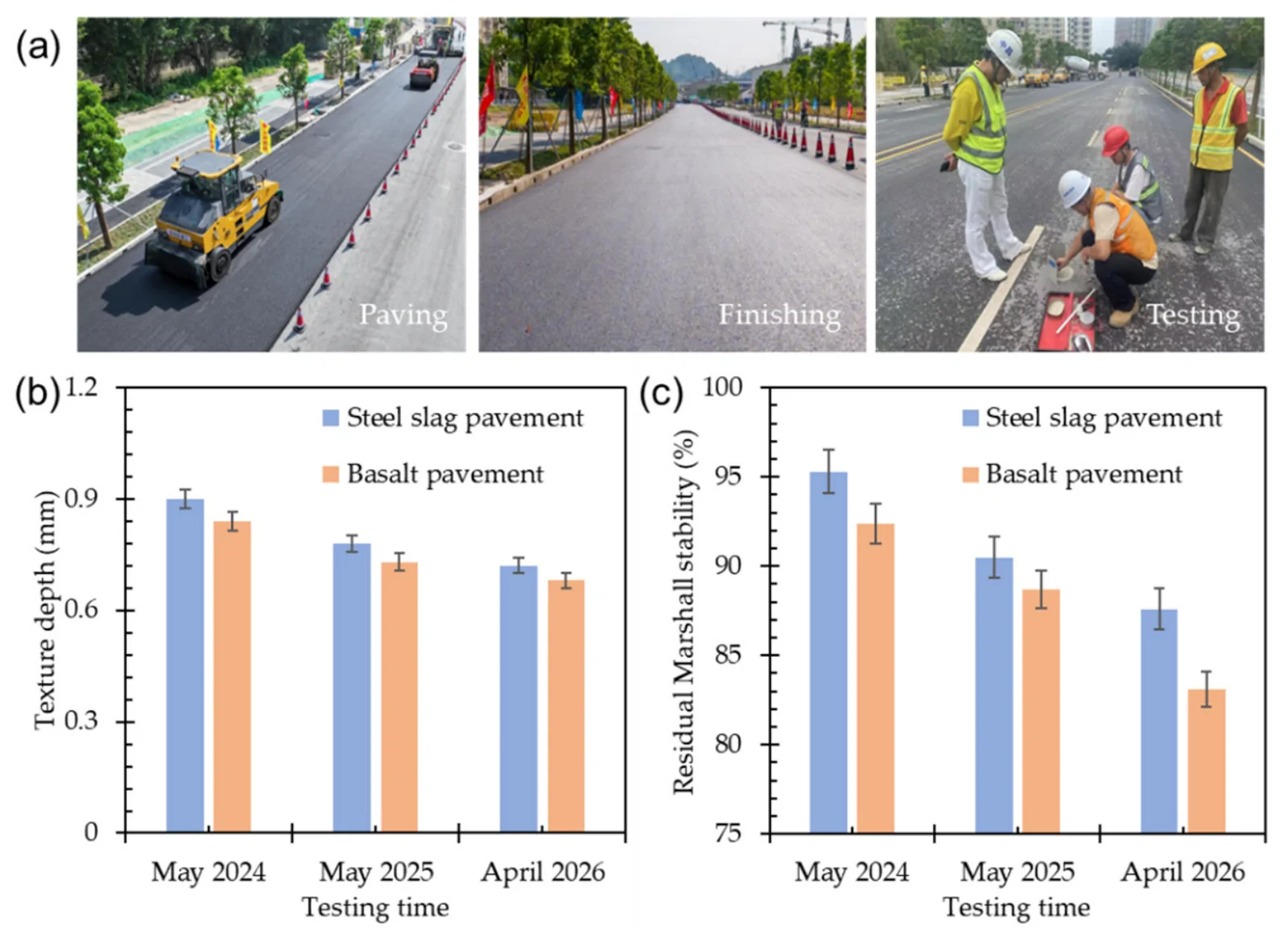
Service performance of steel slag asphalt pavement: (**a**) paving and testing; (**b**) texture depth; (**c**) residual Marshall stability.

Figure 22a shows the construction and detection process of the asphalt pavement. Both mixtures were produced according to the material proportions described in Section 2.2. The mixing, paving, and compaction temperatures were controlled at 180 ± 3 °C, 172 ± 5 °C, and 166 ± 5 °C, respectively. The pavement performance at different service stages was evaluated in accordance with the Field Test Methods of Highway Subgrade and Pavement (JTG 3450-2019) [35]. Specifically, the texture depth was measured periodically using the standard sand-patching method, while the retained Marshall stability was determined through core samples extracted from the pavement and subsequently tested in the laboratory under standard conditions.

Figure 22b,c present the evolution of the texture depth and residual Marshall stability, respectively, over the two-year service period. The field data indicates that the steel slag pavement demonstrated a sustained advantage in both surface macrotexture retention and moisture stability. Specifically, the initial texture depth of the steel slag pavement was 0.82 mm and was maintained at 0.75 mm after two years of service, achieving a remarkable performance retention rate of 91.5%, which notably outperforms the 84.6% retention observed in the basalt pavement. Concurrently, core samples extracted after two years revealed a residual Marshall stability of 87.6% for the steel slag pavement. In contrast, the basalt pavement yielded a value of 83.1%, falling short of the stringent requirements for moisture damage resistance. These field application results conclusively demonstrate that steel slag asphalt pavements possess immense potential for widespread deployment in high-temperature and high-humidity climatic regions.

## 5. Conclusions

To systematically evaluate the performance degradation and failure mechanisms of asphalt pavement materials under combined damp-heat conditions, this study examined steel slag and basalt aggregates and their corresponding asphalt mixtures. Laboratory aging simulations (60–80 °C, 90% RH, 0–8 days) were applied, and a multi-scale characterization was performed—spanning macro-scale physical and rheological properties, micro-scale chemical functional group evolution, and asphalt–aggregate interfacial adhesion behavior. Moreover, by integrating an improved multi-dimensional evaluation model with a two-year field test in a typical damp-heat region, the early-to-mid-term service durability of steel-slag asphalt pavement was validated. The main findings are summarized as follows:

(1) The damp-heat aging of asphalt binders is fundamentally a synergistic deterioration process involving early-stage physical moisture intrusion and long-term thermal-oxidative chemical reactions. Within this complex degradation system, the aging temperature governs the kinetic rates of moisture diffusion and oxidative reactions, playing a more decisive role in accelerating material embrittlement than the exposure duration. Specifically, the creep rate (m-value) of the base asphalt aged at 80 °C for only 2 days declined to 0.281, which surpassed the degradation level observed after 4 days of aging at 60 °C.

(2) Distinct from the continuous and monotonic performance degradation observed in base asphalt under damp-heat conditions, SBS-modified asphalt exhibits a characteristic two-stage aging behavior consisting of initial softening followed by hardening. The three-dimensional cross-linked structure of the SBS polymer network effectively retards moisture penetration and preferentially consumes oxygen to delay the deep oxidation of the asphalt matrix. This enables the binder to maintain superior rheological properties even under extreme damp-heat conditions. Under severe aging at 80 °C for 8 days, the modulus aging index (MAI) of the SBS-modified asphalt was only 20.59%, lower than the 53.09% recorded for the base asphalt, demonstrating exceptional resistance to damp-heat aging.

(3) Steel slag aggregates fundamentally reconstruct the interfacial moisture damage resistance mechanism of asphalt mixtures. Unlike basalt aggregates, which possess smooth surfaces rich in acidic silicon dioxide, the rough and porous micromorphology of steel slag establishes deep mechanical interlocking with the asphalt binder. And its high concentration of alkaline constituents is hypothesized to facilitate a chemical anchoring effect through chemical reactions with acidic components in asphalt. The adhesion property of steel slag following 8 days of damp-heat aging improved by 27.0% compared to that of basalt. Additionally, the SBS-modified steel slag mixture achieved an indirect tensile strength of 1.118 MPa, representing a 29.2% strength advantage over the basalt-based counterpart.

(4) The trajectory of damp-heat aging evolution adheres to the time–temperature superposition principle, and the incorporation of a steel slag skeleton noticeably reduces the time–temperature sensitivity of the mixture to damp-heat environments. Comprehensive evaluation using the improved radar chart indicates that under extreme aging conditions, the comprehensive index (f-value) of the basalt mixture was only 0.1949, reflecting a 79.0% decay. In contrast, the steel slag mixture sustained an f-value of 0.3151. Following two years of service in high-temperature and high-humidity regions, the steel slag asphalt pavement maintained a residual Marshall stability of 87.6% and a texture depth retention rate of 91.5%. These findings demonstrate that steel slag asphalt mixtures provide a reliable material solution for enhancing the durability of pavements in damp-heat climatic zones.

## 6. Perspectives for Future Research

This study focused on the effects of different damp-heat aging temperatures and durations on the performance of steel slag and basalt asphalt mixtures, as well as the underlying mechanisms. The experimental results preliminarily suggest that the impact of damp-heat aging on asphalt mixture performance may follow the time–temperature superposition principle, although more rigorous validation is needed in the future. Future research could also consider extending the humidity range and exploring different steel slag contents.

## Figures and Tables

**Table 1:** Property of 60/80 asphalt and SBS-modified asphalt.

Asphalt Binder	Properties	Results
60/80 asphalt	Penetration (25 °C, 100 g, 5 s; 0.1 mm)	73.5
	Ductility (15 °C; cm)	102.3
	Softening point (°C)	46.5
	Viscosity (135 °C; mPa·s)	448
After TFOT	Penetration (25 °C, 100 g, 5 s; 0.1 mm)	51.5
	Ductility (15 °C; cm)	68.7
	Softening point (°C)	53.1
SBS modified asphalt	Penetration (25 °C, 100 g, 5 s; 0.1 mm)	52.8
	Ductility (5 °C; cm)	36.3
	Softening point (°C)	68.2
	Viscosity (135 °C; mPa·s)	1853
After TFOT	Penetration (25 °C, 100 g, 5 s; 0.1 mm)	44.9
	Ductility (5 °C; cm)	22.4
	Softening point (°C)	75.8

**Table 2:** Property of steel slag aggregate and basalt.

Technical Index		Tested Values	
		Steel Slag	Basalt
Apparent specific gravity	10–16 mm	3.473	2.713
	5–10 mm	3.481	2.725
	0–5 mm	3.595	2.728
Water absorption (%)	10–16 mm	1.3	0.4
	5–10 mm	1.4	0.5
	0–5 mm	1.5	0.9
Crushing value (%)	-	12.9	13.3
f-CaO content (%)	10–16 mm	1.2	/
	5–10 mm	0.8	/
	0–5 mm	0.7	/

**Table 3:** Property of mineral filler.

Technical Index		Mineral Filler
Apparent specific gravity		2.719
Particle size range (%)	<0.6mm	100
	<0.15mm	98.2
	<0.075mm	95.5
Hydrophilic coefficient		0.80

**Table 4:** Aggregate gradation information.

Screen Size (mm)		16	13.2	9.5	4.75	2.36	1.18	0.6	0.3	0.15	0.08
Passing rate of basalt aggregate (%)	10–16 mm	100	90.1	23.5	0.1	0.1	0.1	0.1	0.1	0.1	0.1
	5–10 mm	100	100	100	15.2	0.1	0.0	0.0	0.0	0.0	0.0
	0–5 mm	100	100	100	99.2	69.6	48.3	32.3	16.0	9.1	4.1
Passing rate of steel slag aggregate (%)	10–16 mm	100	93.8	45.7	3.7	1.3	1.3	1.3	1.3	1.3	0.9
	5–10 mm	100	99.1	88.2	23.1	2.4	2.2	2.1	2.1	2.1	1.6
	0–5 mm	100	100	100	99.5	80.7	61.1	47.8	30.8	21.7	14.3
Passing rate of mineral filler		100	100	100	100	100	100	100	100	98.2	95.5

**Table 5:** Mineral aggregate composition of steel slag and basalt asphalt mixture.

Aggregate Type		Content (%)	
		Basalt Asphalt Mixture	Steel Slag Asphalt Mixture
Basalt aggregate	10–16 mm	28	/
	5–10 mm	27	/
	0–5 mm	40	/
Steel slag aggregate	10–16 mm	/	40
	5–10 mm	/	21
	0–5 mm	/	39
Mineral filler		5	/

**Table 6:** Aggregate gradation of steel slag and basalt asphalt mixture.

Screen Size (mm)	16	13.2	9.5	4.75	2.36	1.18	0.6	0.3	0.15	0.08
Passing rate of basalt aggregate (%)	100	97.2	78.6	48.8	32.9	24.4	18.0	11.4	8.6	6.5
Passing rate of steel slag aggregate (%)	100	97.3	75.8	45.1	32.5	24.8	19.6	13.0	9.4	6.3

**Table 7:** Volume parameters of steel slag and basalt asphalt mixture.

Technical Index	Asphalt Mixture Type		Specification Limits [28]
	Basalt Asphalt Mixture	Steel Slag Asphalt Mixture	
Maximum theoretical specific gravity	2.529	3.125	/
Bulk density (g/cm^3^)	2.422	2.983	/
Void ratio (%)	4.2	4.5	3~6
VMA (%)	15.1	16.0	≥14
VFA (%)	71.9	71.6	65~75
Marshall stability (kN)	14.5	15.6	≥8
Flow value (mm)	3.2	2.7	2~4

**Table 8:** Damp-heat aging parameters of base asphalt and SBS modified asphalt.

Number	Temperature (°C)	Asphalt Type	Aging Time (d)	Sample ID
1	60	Base asphalt	0, 2, 4, 6, 8	B-60
2	70			B-70
3	80			B-80
4	60	SBS modified asphalt		S-60
5	70			S-70
6	80			S-80

**Table 9:** Damp-heat aging parameters of asphalt mixture.

Number	Temperature (°C)	Asphalt Type	Aggregate Type	Aging Time (d)	Sample ID
1	60	Base asphalt	Steel slag	0, 2, 4, 6, 8	B-SM-60
2	70				B-SM-70
3	80				B-SM-80
4	60	SBS modified asphalt			S-SM-60
5	70				S-SM-70
6	80				S-SM -80
1	60	Base asphalt	Basalt	0, 2, 4, 6, 8	B-BM-60
2	70				B-BM-70
3	80				B-BM-80
4	60	SBS modified asphalt			S-BM-60
5	70				S-BM-70
6	80				S-BM -80

**Table 10:** Surface elemental composition of basalt aggregates.

Element	wt%	wt% Sigma
C	11.37	0.49
O	37.98	0.24
Mg	4.55	0.04
Al	4.96	0.04
Si	14.98	0.09
Ca	3.76	0.03
Ti	3.07	0.03
Mn	0.17	0.03
Fe	19.17	0.13
Total content	100	

**Table 11:** Surface elemental composition of steel slag.

Element	wt%	wt% Sigma
C	13.83	0.15
O	40.84	0.12
Mg	1.54	0.02
Al	1.69	0.02
Si	4.59	0.03
Ca	28.62	0.08
Mn	1.79	0.04
Fe	7.09	0.06
Total content	100	

## Data Availability

The original contributions presented in this study are included in the article. Further inquiries can be directed to the corresponding authors.

## References

[1] Song Y., Feng J., Wang F., Wu S., Xu H. (2026). Advanced and Sustainable Approach for Large-Scale, High-Quality Recycling of Predominant Pavement Waste and Its Life Cycle Environmental Impact Assessment. Environ. Impact Assess. Rev..

[2] Xu H., Zou Y., Airey G., Wang H., Zhang H., Wu S., Chen A. (2024). Wetting of Bio-Rejuvenator Nanodroplets on Bitumen: A Molecular Dynamics Investigation. J. Clean. Prod..

[3] Chen Q., Ge T., Li X., Li X., Wang C. (2025). Road Area Pollution Cleaning Technology for Asphalt Pavement: Literature Review and Research Propositions. J. Clean. Prod..

[4] Yan X., Wu D., Hu K., Zhang W., Xing J., Cui L., Shi S., Yang J., Yang C. (2023). The Modification Mechanism, Evaluation Method, and Construction Technology of Direct-to-Plant SBS Modifiers in Asphalt Mixture: A Review. Polymers.

[5] Coelho L.M., dos Santos W.W., Guimaraes A.C.R., Monteiro S.N. (2024). Influence of the Bailey Gradation Method on the Mechanical Behavior of Asphalt Mixture Containing Steel Slag as an Alternative Aggregate. Buildings.

[6] Xu G., Yao Y., Wu M., Zhao Y. (2023). Molecular Simulation and Experimental Analysis on Co-Aging Behaviors of SBS Modifier and Asphalt in SBS-Modified Asphalt. Mol. Simul..

[7] Zhao W., Fang G., Qin X., Mao J. (2024). Multiscale Characterization of the UV Aging Resistance and Mechanism of Light Stabilizer-Modified Asphalt. Rev. Adv. Mater. Sci..

[8] Abouelsaad A., White G. (2022). Comparing the Effect of Thermal-Oxidation and Photo-Oxidation of Asphalt Mixtures on the Rheological and Chemical Properties of Extracted Bituminous Binder. Materials.

[9] Pei Z., Xu M., Cao J., Feng D., Hu W., Ren J., Jing R., Yi J. (2022). Analysis of the Microcharacteristics of Different Kinds of Asphalt Based on Different Aging Conditions. Mater. Struct..

[10] Song Y., Lu Z., Feng J., Wu S., Wan P., Gong X., Xu H., Xie J. (2026). Investigation on the Performance, Environmental and Economic Benefits of Coarse-Grained Recycled Asphalt Mixture with High RAP Content. Constr. Build. Mater..

[11] Zhen T., Kang X., Liu J., Zhang B., Si W., Ling T. (2023). Multiscale Evaluation of Asphalt Aging Behaviour: A Review. Sustainability.

[12] Abraham S.M., Verma M., Kakade V. (2023). Aging Resistance of Bitumen Modifiers: A Comprehensive Review. J. Test. Eval..

[13] Liu Q., Li J., Chen S. (2024). Effects of Different Antioxidant Intercalated Layered Double Hydroxides on Anti-Aging Properties of Asphalt Binders. Buildings.

[14] Luo Y., Zhang H., Chen Z., Li Q., Ye S., Liu Q. (2024). Novel Multidimensional Composite Development for Aging Resistance of SBS-Modified Asphalt by Attaching Zinc Oxide on Expanded Vermiculite. Energy Fuels.

[15] (2004). Technical Specifications for Construction of Highway Asphalt Pavements.

[16] Cheng Y., Wang H., Li L., Liang J. (2023). A Multi-Scale Study of SBS Modified Asphalt-Aggregate Adhesion Under Moisture Damage. Arab. J. Sci. Eng..

[17] Lu J., Gong M., Chen J., Zhang D., Liu Z. (2021). Study on the Decay Behavior of the Stability of Asphalt Mixture under Different Dry-Wet Cycle Conditions. Constr. Build. Mater..

[18] Maurya A.K., Singh D. (2024). Novel Approach to Characterize Moisture Damage in Asphalt Mixtures Based on Critical Permeability. J. Mater. Civ. Eng..

[19] Song Y., Feng J., Wu S., Xu H., Tu L., Xie J. (2025). Effect of Marshall Compaction Parameters on the Performance and Internal Structure of Steel Slag Asphalt Concrete. J. Clean. Prod..

[20] Liu S., Shan L., Li G., Underwood B.S., Qi C. (2022). Molecular-Based Asphalt Oxidation Reaction Mechanism and Aging Resistance Optimization Strategies Based on Quantum Chemistry. Mater. Des..

[21] Ma X., Zhang C., Li R., Zheng L., Xu J. (2025). Effect of Temperature and Humidity Environment on the Microstructure and Physicochemical Properties of SBS Modified Asphalt. Case Stud. Constr. Mater..

[22] Akentuna M., Liu J., Mohammad L.N., Sachdeva S., Cooper S.B., Cooper S.B. (2025). Moisture Susceptibility of Asphalt Mixtures: Conditioning and Testing Protocols. Transp. Res. Rec..

[23] Das B.P., Siddagangaiah A.K. (2022). Moisture Damage Analysis Based on Adhesive Failure in Asphalt Mixtures. Int. J. Pavement Eng..

[24] Ren M., Han Q., Wang L., Xue Z., Li T., Li X. (2025). Fracture Modeling of Steel Slag Asphalt Mixtures Considering Moisture-Induced Slag Expansion under Long-Term Vapor Exposure. Constr. Build. Mater..

[25] Wang L., Xue Z., Zhang L. (2022). Effect of Thermal-Oxygen Aging on Temperature Stability of Styrene-Butadiene-Styrene-Modified Asphalt. J. Test. Eval..

[26] Zhang H., Chen Z., Xu G., Shi C. (2018). Evaluation of Aging Behaviors of Asphalt Binders through Different Rheological Indices. Fuel.

[27] Hou X., Lv S., Chen Z., Xiao F. (2018). Applications of Fourier Transform Infrared Spectroscopy Technologies on Asphalt Materials. Measurement.

[28] Ministry of Transport of the People’s Republic of China (2004). Technical Specification for Construction of Highway Asphalt Pavements.

[29] Ministry of Transport of the People’s Republic of China (2025). Standard Test. Methods of Asphalt and Asphalt Mixture for Highway Engineering.

[30] Song Y., Wu S., Chen A., Li Y. (2023). Physical, Rheological, and Anti-Ultraviolet Aging Performance of Layered Double Hydroxides plus Styrene Block Copolymer-Modified Asphalt Binders. Sustainability.

[31] Zhao P., Wang C., Liang H., Zhang W., Fan L., Cao F., Zhou X., Ren H. (2024). Identification of Aging Fingerprint of Base Asphalt by ATR-FTIR Combined with Chemometrics. Road Mater. Pavement Des..

[32] Gao Y., Gu F., Zhao Y. (2013). Thermal Oxidative Aging Characterization of SBS Modified Asphalt. J. Wuhan. Univ. Technol.-Mat. Sci. Ed..

[33] Jiang Y., Xu T. (2024). Microscopic Insights into the Long-Term Ageing Mechanism of Asphalt in the Hygrothermal Environments. Mater. Chem. Phys..

[34] Xu H., Wu S., Wang H., Xie J., Yang X., Song Y., Feng J., Zhu Y. (2026). Interfacial Behavior and Molecular Insights into Adhesion between Metallurgical Slag and Bitumen for Sustainable Asphalt Pavements. Constr. Build. Mater..

[35] Ministry of Transport of the People’s Republic of China (2019). Field Test Methods of Highway Subgrade and Pavement.

